# High gain differentiator based neuro-adaptive arbitrary order sliding mode control design for MPE of standalone wind power system

**DOI:** 10.1371/journal.pone.0293878

**Published:** 2024-01-18

**Authors:** Ammar Ali, Qudrat Khan, Safeer Ullah, Asad Waqar, Lyu-Guang Hua, Imen Bouazzi, Liu Jun Jun

**Affiliations:** 1 Department of Electrical Engineering, Bahria University, Islamabad, Pakistan; 2 Centre for Advanced Studies in Telecommunications (CAST), COMSATS University, Islamabad, Pakistan; 3 Department of Electrical Engineering, Quaid-e-Azam College of Engineering & Technology, Sahiwal, Pakistan; 4 Power China Huadong Engineering Co. Ltd, Hang Zhou, China; 5 Department of Industrial Engineering, College of Engineering, King Khalid University, Abha, Saudi Arabia; Huazhong University of Science and Technology, CHINA

## Abstract

In this paper, we introduce a novel Maximum Power Point Tracking (MPPT) controller for standalone Wind Energy Conversion Systems (WECS) with Permanent Magnet Synchronous Generators (PMSG). The primary novelty of our controller lies in its implementation of an Arbitrary Order Sliding Mode Control (AOSMC) to effectively overcome the challenges caused by the measurement noise in the system. The considered model is transformed into a control-convenient input-output form. Additionally, we enhance the control methodology by simultaneously incorporating Feedforward Neural Networks (FFNN) and a high-gain differentiator (HGO), further improving the system performance. The FFNN estimates critical nonlinear functions, such as the drift term and input channel, whereas the HGO estimates higher derivatives of the system outputs, which are subsequently fed back to the control inputs. HGO reduces sensor noise sensitivity, rendering the control law more practical. To validate the proposed novel control technique, we conduct comprehensive simulation experiments compared against established literature results in a MATLAB environment, confirming its exceptional effectiveness in maximizing power extraction in standalone wind energy applications.

## 1 Introduction

Increasing population, economic development, and energy demand have led to the establishment of new power plants to meet growing needs. However, the limitations imposed by the energy crisis, higher oil prices, and climate change have emphasized the importance of Renewable Energy Resources (RESs) [1]. Globally, governments have focused on effective and environmentally friendly sustainable renewable energy systems. The power sector relies heavily on hydro and gas power stations, but there is a need to diversify and catch up with the growing demand. Rural and urban areas located far from the grid supply can benefit from renewable energy sources. Fossil fuels used for electricity generation are becoming scarce, prompting governments to prioritize a secure and sustainable energy economy. Solar and wind energy systems have witnessed substantial global growth with annual growth rates of 25–30% over the past decade [2, 3]. These advancements in RESs are crucial for addressing energy demand while considering environmental concerns and the finite nature of non-renewable energy sources.

Wind energy is a green and environmentally friendly resource that offers a solution for reducing dependence on fossil fuels and alleviating their adverse environmental effects, as indicated by Dali et al. [4]. Wind power system efficiency has a significant impact on industrial and commercial power sectors. As wind energy has rapidly emerged as a competitive renewable energy source, it has several advantages, including abundant availability and minimal adverse environmental effects. Harnessing wind energy involves a mechanical-to-electrical energy transformation using wind turbines (WTs).

In recent decades, the installed capacity of wind energy has grown exponentially, making it a viable option for increasing the penetration of renewable energy. Technological advancements have contributed significantly to various aspects of the wind energy industry, including power electronic converters, aerodynamic design, mechanical systems, control theory, and power system integration. Electric generators, control theory, and power electronic converters play crucial roles in enabling a WECS to operate safely, reliably, and efficiently, while meeting stringent grid code requirements. WECS holds high priority among renewable energy sources due to its significant output energy potential. As a result, extracting the maximum power (MP) from wind power systems (WPS) has become a prominent research area. Wind-speed-sensorless MPPT control has attracted considerable attention in academia. MPPT is a control technique employed in wind turbines to maximize power extraction under various climatic conditions [5]. This is achieved without physically moving the wind turbine or the other system components. Although MPPT can be used with a mechanical framework, the two systems are distinct. The literature offers insights into MPPT techniques and design considerations for WPS [6–8].

In most studies, robust controllers have been developed and studied (e.g., [9–19]) for the extraction of maximum power *P*_*max*_ from the wind. Majid and Yatim [20] studied a technique for the extraction of reactive power and maximum inverter power using the variable-speed WT modulation index (*ma*) and the terminal voltage power angle (*δ*) of an inverter without a wind speed sensor. Francoise et al. [21] carried out an adaptive MPPT scheme while using variable wind speed to a PMSG, which is further attached to a battery charging station. Nicholas et al. in [22] investigate the performance of a full-variable wind turbine that a nonlinear backstepping controller controlled. Lyapunov analysis shows that this controller is stable while achieving the desired generator rotational speed. Some drawbacks include a higher steady-state error and a lower dynamic response. Lin and Chengpeng [23] investigated and designed an improved sliding mode control for a WECS, a grid-connected offshore wind turbine PMSG. By employing an aerodynamic torque observer, the performance of the MPPT system can be significantly improved, effectively addressing the chattering issues arising from the variable nature of the wind speed in wind energy systems. In [24, 25], the authors introduce a strategy aimed at tackling this issue. Their approach integrates integral action to eliminate steady-state errors and employs adaptive control to dynamically adjust control gains in real-time. Janusz and Andrzej [26] performed a simulation study to analyze a variable-speed fixed-pitch WECS equipped with a three-phase PMSG under diverse wind conditions. This study focused on the utilization of a linear disturbance observer and feedforward control to compensate for wind turbine aerodynamic torque estimation. This study offers valuable insights into the operational zones of a WECS MPPT system for different wind-speed profiles. However, it is important to note that the simulation model did not account for electrical phenomena such as short circuits or voltage dips occurring in the WECS-grid connection. Consequently, the model may not fully adhere to certain standard requirements.

In addition to the aforementioned techniques, numerous other MPPT control techniques have been developed to maximize power extraction from wind energy systems, regardless of the generator employed in the WECS. To enhance the efficiency, profitability, and reliability of a WECS, these innovative control techniques have emerged as pivotal factors [27]. MPPT techniques can be broadly classified into three categories: conventional, population-based, and artificial intelligence (AI) techniques. For variable-speed generator systems, adjusting the optimal tip ratio to its optimal value enables effective tracking of the MPP even under varying wind speeds [28]. Maintaining the maximum power output across all wind speeds poses a challenge because of the nonlinear characteristics inherent in wind turbines [29, 30]. Consequently, various techniques have been employed to estimate the MP of wind-turbine systems. Some methods utilize power generation changes to identify the maximum power point. However, these methods often rely on mechanical sensors to measure generator speed, which may introduce vulnerabilities owing to modeling inaccuracies and insensitivity in certain scenarios [31–34].

The present research work presents significant and prominent contributions, outlined as follows:

1. The central focus of this study is the introduction of a control methodology tailored explicitly for standalone PMSG-WECS, addressing the presence of measurement noise. The primary goal of the finite-time concept in the proposed SMC is to achieve stability within a predetermined time precisely and track the maximum power point for optimizing power output.
2. The paper dedicates significant attention to transforming the dynamic model of the considered PMSG-WECS system into a control-convenient input-output format. This transformation forms the foundational basis for the subsequent design of the control methodology.
3. A significant advancement in this research involves the integration of advanced techniques into the control methodology. Specifically, FFNN are employed for estimating crucial nonlinear functions, including the nonlinear drift term and the input channel. Concurrently, HGO techniques are used to estimate the higher derivatives of system outputs. These estimations are seamlessly incorporated into the control inputs, resulting in a reduced sensitivity to sensor noise. This enhancement substantially bolsters the practicality of the control law, enabling it to effectively manage the inevitable measurement noise encountered in real-world systems.
4. Finite-time techniques are designed to establish stability within a defined time frame, enabling fast control responses. On the other hand, high-gain differentiators are employed for accelerated state estimation, noise resilience, and disturbance tolerance. The integration of these approaches synergistically elevates control system performance, particularly in critical applications such as wind energy systems, where the need for fast responses and noise robustness is paramount.
5. To comprehensively validate the proposed control technique, extensive simulation experiments are conducted within a MATLAB environment. The results of these simulations undergo rigorous benchmarking against well-established literature results [35, 36], providing compelling evidence of the effectiveness and prowess of the proposed control design.

The remainder of this paper is organized as follows. Section 2 describes the equivalent model of a PV array accompanied by underlying mathematical concepts. In Section 3, a control algorithm that synergizes with a feedforward neural network and high-gain differentiator to attain optimal power extraction is formulated. The exposition of the simulation outcomes, which vividly demonstrate the robustness of the introduced controller, is presented in Section 4. Finally, Section 5 provides concluding remarks for this research endeavor.

## 2 Modeling of the wind turbine systems

**Fig 1:**
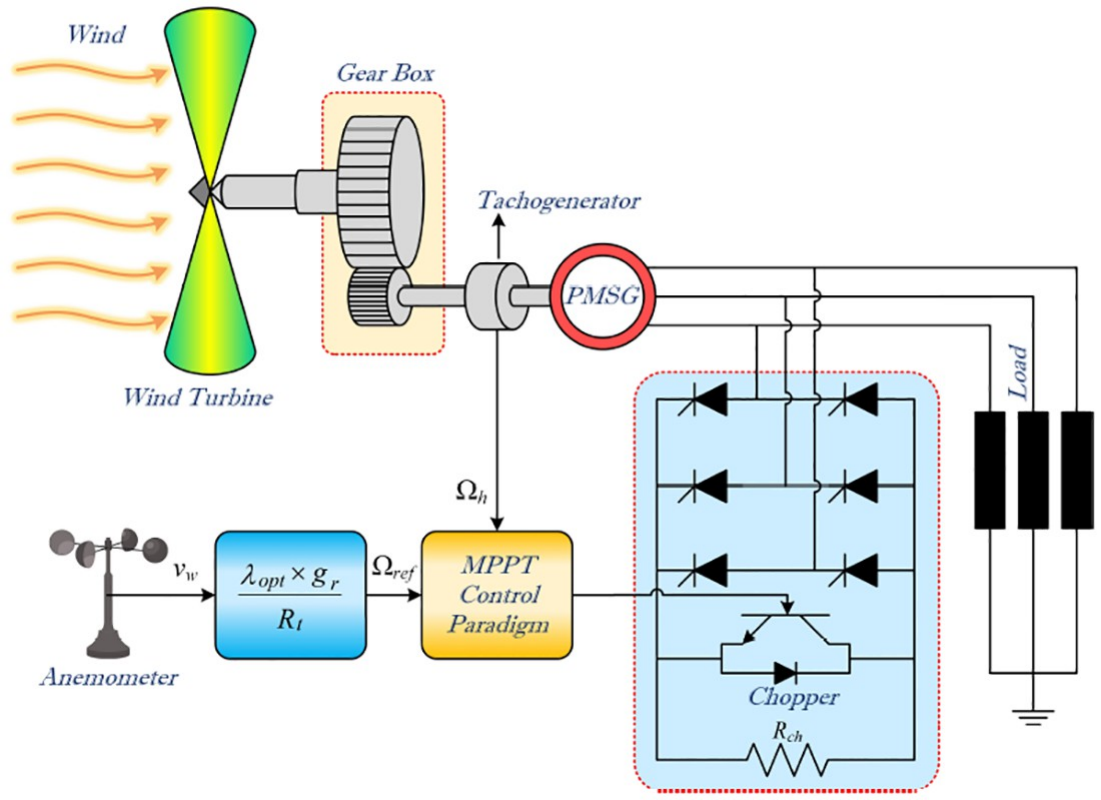
The wind energy conversion system built around a PMSG.

**Fig 2:**
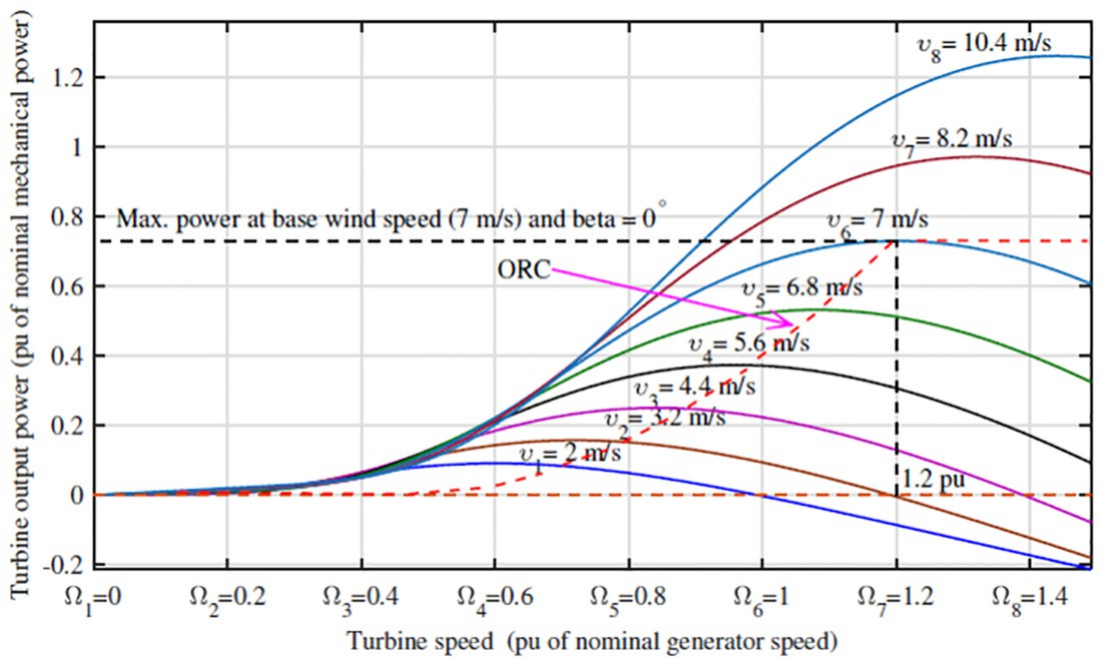
Turbine speed vs turbine output mechanical power.

To understand the behavior and optimize the performance of a wind turbine, it is crucial to develop a mathematical model that describes its operation within a specific operating range. Fig 1 illustrates the complete physical process of a WECS [37]. The generation of mechanical power begins with airfoil lift, which creates a positive torque on the rotating shaft. The generator converts mechanical power into electrical power. The interaction between the rotor and the wind at the core of this process is shown in Fig 2. The mean wind speed directly affects the mean output power, emphasizing the importance of considering steady-state aerodynamics while neglecting turbulence [38]. Understanding the key characteristics of wind turbine performance, such as power production and loads influenced by wind, is essential.

In the following subsections, the dynamics of the rotor blade, and synchronous generators are briefly discussed in order to design the control technique.

### 2.1 Dynamic model of the turbine’s rotor blade

Aerodynamic analysis and modeling of variable-speed wind turbines have been extensively studied in the literature (see [39, 40] for further details). Betz conducted work on wind turbine aerodynamics from the 1920s to the 1930s [39]. A review of an ideal wind-turbine configuration is presented in [20], which considered a rotor with multiple blades capable of extracting a maximum power of 59.26% under ideal conditions. However, in practical scenarios, this value typically falls close to 50% owing to factors such as the number of blades and rapid changes in the external environment.

The wind power generated by a wind turbine can be mathematically expressed as

$$\begin{array}{c} P_{M}=\frac{1}{2}\rho AV_{\text{wind}}^{3} \end{array}$$ (1)

where *P*_*M*_ represents the mechanical power output of the turbine, *A* is the area swept by the blades, *ρ* is the air density, and *V*_wind_ is the wind speed (m/s). The wind turbine rotor power-conversion coefficient, denoted by *C*_*p*_(λ, *β*), represents the ratio of extractable power to available power. It is modeled in [41] by the following equation

$$\begin{array}{c} P_{M}=\frac{1}{2}\rho \pi {R_{t}}^{2}V_{\text{wind}}^{3}C_{p}\left(\lambda ,\beta \right) \end{array}$$ (2)

where *R*_*t*_ represents the blade radius (m), *β* is the blade pitch angle, and λ is the tip-speed ratio (TSR). The TSR is defined as the ratio of the peripheral speed of the wind turbine blades to the wind speed and can be defined as

$$\begin{array}{c} \lambda =\frac{R_{t}\omega_{t}}{V_{\text{wind}}} \end{array}$$ (3)

where *V*_wind_ denotes the wind speed, *ω*_*t*_ denotes the rotor speed, and *R*_*t*_ denotes the rotor radius. Power coefficient *C*_*P*_ is a function of λ and *β*. To maximize power extraction from the wind, the tip speed ratio should operate at its optimum value, λ_opt_ = 7. At this optimal TSR, the rotor shaft speed precisely follows the reference speed *ω*_ref_ calculated using the following formula:

$$\begin{array}{c} \omega_{\text{ref}}=\frac{\lambda_{\text{opt}}V_{\text{wind}}}{R_{t}} \end{array}$$ (4)

**Fig 3:**
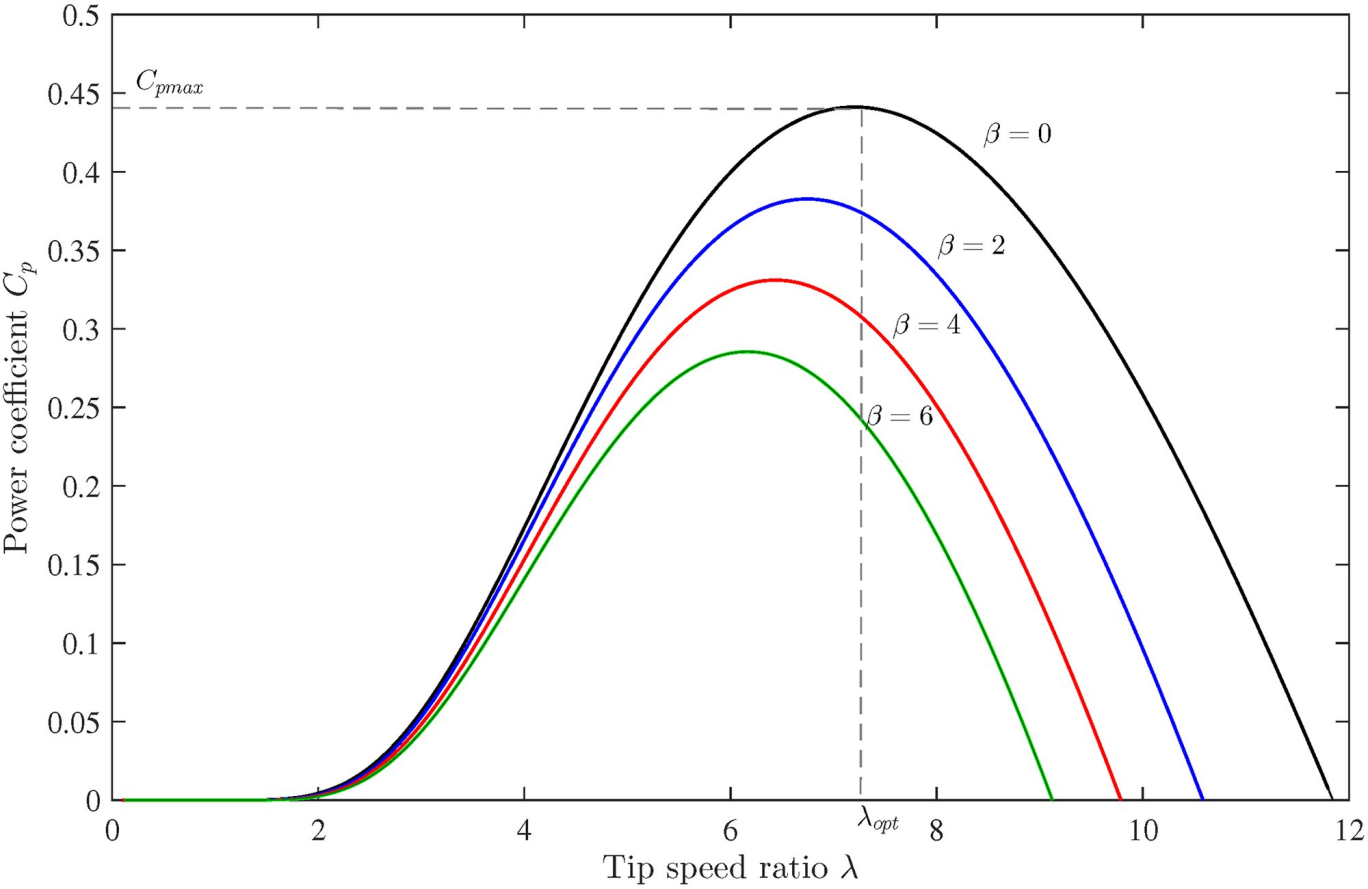
Variation of power coefficient with tip speed ratio for different pitch angles.

For a variable-speed wind turbine, an approximation for *C*_*p*_ can be determined using the coefficients *a*_0_, *a*_1_, *a*_2_, *a*_3_, *a*_4_, *a*_5_, *a*_6_, and *a*_7_ as follows [42]

$$\begin{array}{c} C_{p}\left(\lambda \right)=a_{0}+a_{1}\lambda +a_{2}\lambda^{2}+a_{3}\lambda^{3}+a_{4}\lambda^{4}+a_{5}\lambda^{5}+a_{6}\lambda^{6}+a_{7}\lambda^{7} \end{array}$$ (5)

where *a*_0_ = 0, *a*_1_ = 0.0061, *a*_2_ = −0.0013, *a*_3_ = 0.0081, *a*_4_ = −0.000974, *a*_5_ = 0.0000654, *a*_6_ = 0.00000130, and *a*_7_ = −0.000000454. The power coefficient *C*_*p*_ versus TSR graph for different pitch angles is shown in Fig 3 [43]. It is worth emphasising that wind speed can be represented by the following expression:

$$\begin{array}{c} V_{\text{wind}}=R_{t}\omega_{M} \end{array}$$

Substituting this expression into Eq (2), we obtain

$$\begin{array}{c} P_{M}=\frac{1}{2}\rho \pi {R_{t}}^{2}{\left(R\omega_{M}\right)}^{3}C_{p}\left(\lambda ,\beta \right) \end{array}$$ (6)

where $\frac{1}{2}\rho R_{t}^{2}$ can be represented as a constant, denoted by *K*_con_. When *β* = 0, Eq (6) simplifies to

$$\begin{array}{c} P_{M}=K_{\text{con}}{\left(\omega_{M}\right)}^{3}C_{p}\left(\lambda \right) \end{array}$$ (7)

The aerodynamic power of a PMSG can be mathematically represented as follows

$$\begin{array}{c} P_{M}=\Gamma_{\text{wind}}\omega_{t} \end{array}$$ (8)

where *ω*_*t*_ is defined as

$$\begin{array}{c} \omega_{t}=\lambda V_{\text{wind}}/R_{t} \end{array}$$ (9)

Table 1 lists all the necessary wind-turbine parameters used in this study.

**Table 1:** Parameters of the wind turbine.

Parameters	Symbol	Value	Units
Air density	*ρ*	1.25	*kg*/*m*^3^
Gears Ratio /Transmission Ratio	*i*	7	
Blade Radius	*R*_*t*_	2.5	M
Maximum Power Coefficient	$C_{p_{max}}$	0.47	
Optimal Tip Speed Ratio	λ_*opt*_	7	
High Speed Shaft Inertia	*j*_*h*_	0.0552	*kg*.*m*^2^

In the following subsection, we will discuss the modeling of the mechanical subsystem.

### 2.2 Modeling of the mechanical subsystem

The mechanical torque of the turbine shaft can be defined as the ratio of the output mechanical power to rotor speed.

$$\begin{array}{c} \text{Turbine}\;\text{mechanical}\;\text{torque}=\frac{\text{Output}\;\text{Mechanical}\;\text{Power}}{\text{Rotor}\;\text{Speed}} \end{array}$$ (10)

This relationship can be expressed as

$$\begin{array}{c} P_{M}=\Gamma_{\text{wind}}\omega_{t} \end{array}$$ (11)

The mechanical power of the shaft, denoted by Γ_wind_, is expressed by

$$\begin{array}{c} \Gamma_{\text{wind}}=0.5\rho \pi R_{t}^{3}V_{w}^{2}C_{T}\left(\lambda \right) \end{array}$$ (12)

where The turbine torque coefficient *C*_*T*_(λ) is defined as the ratio of the power coefficient *C*_*p*_(λ) to the tip speed ratio λ, *ρ* denotes the air density, *R*_*t*_ is the turbine radius, and *V*_*w*_ represents the wind velocity.

### 2.3 Modeling of the electrical subsystem

**Fig 4:**
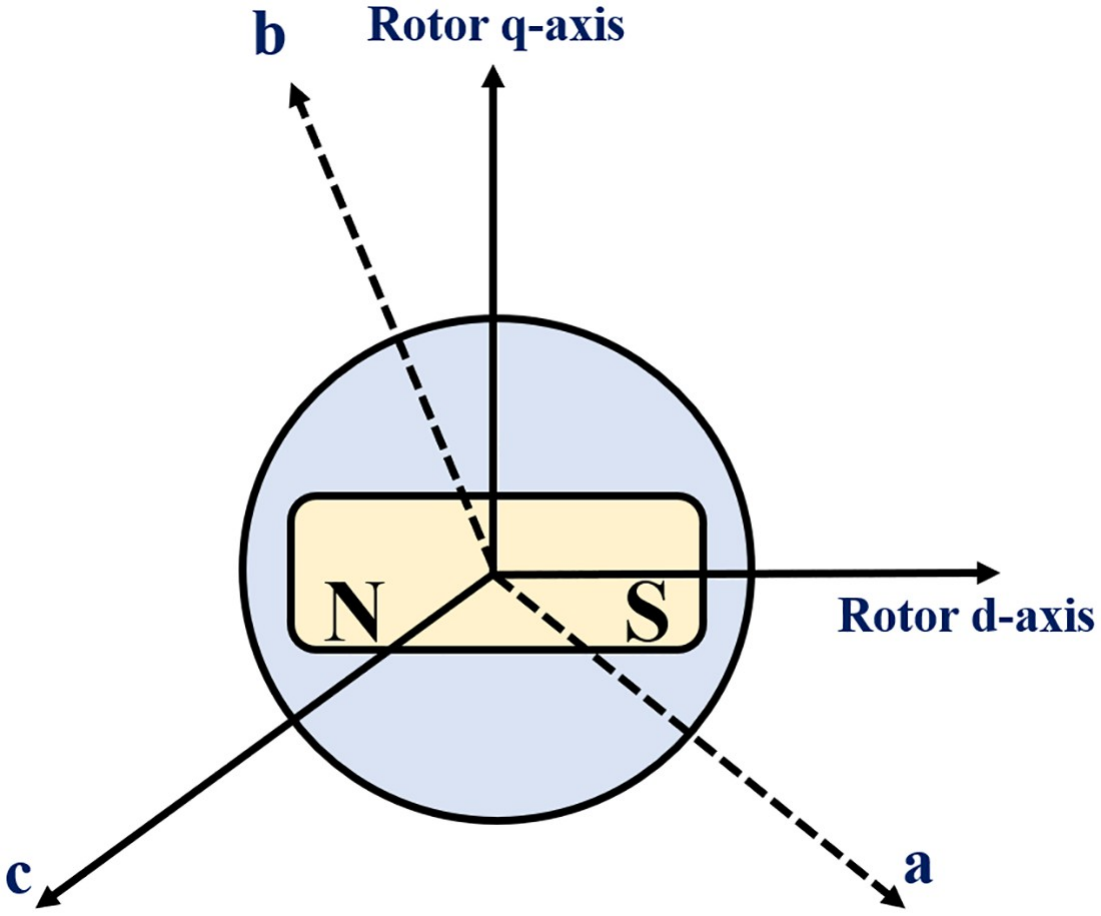
Three phase PMSG with one pole pair permanent magnet.

The dynamics of a three-phase permanent magnet synchronous generator can be simplified using Park’s transformation to reduce complexity. The PMSG consists of three stator windings denoted as *a*, *b*, and *c*. The d-axis corresponds to a winding parallel to the rotor, whereas the q-axis represents a winding perpendicular to the rotor, as shown in Fig 4.

The d-axis current is obtained by summing the contributions from the stator windings projected onto the direct axis, as follows:

$$\begin{array}{c} i_{d}=k_{d}\left[i_{a}\text{cos}\left(\theta \right)+i_{b}\text{cos}\left(\theta -120\right)+i_{c}\text{cos}\left(\theta +120\right)\right] \end{array}$$ (13)

Similarly, the q-axis current is given by

$$\begin{array}{c} i_{q}=k_{q}\left[i_{a}\text{sin}\left(\theta \right)+i_{b}\text{sin}\left(\theta -120\right)+i_{c}\text{sin}\left(\theta +120\right)\right] \end{array}$$ (14)

where *k*_*d*_ and *k*_*q*_ denote arbitrary constants, and *θ* represents the position of the rotor’s direct axis relative to the phase *a* axis. To maintain dimensionality, the zero-sequence current is defined based on Kirchhoff’s current law.

$$\begin{array}{c} i_{0}=k_{0}\left[i_{a}+i_{b}+i_{c}\right] \end{array}$$ (15)

where $k_{d}=k_{q}=\frac{2}{3}$ and $k_{0}=\frac{1}{3}$. Eqs (13), (14) and (15) can be compactly expressed in matrix form, as follows:

$$\begin{array}{c} \left[\begin{array}{c} i_{d}\\ i_{q}\\ i_{0} \end{array}]=\frac{2}{3}[\begin{array}{ccc} \text{cos}(\theta ) & \text{cos}(\theta -120) & \text{cos}(\theta +120)\\ \text{sin}(\theta ) & \text{sin}(\theta -120) & \text{sin}(\theta +120)\\ 1/2 & 1/2 & 1/2 \end{array}][\begin{array}{c} i_{a}\\ i_{b}\\ i_{c} \end{array}\right] \end{array}$$ (16)

The application of Park’s transformation matrix *P* allows for the transformation of currents, voltages, and fluxes. The resulting transformed *dq*0model currents, voltages, and fluxes are expressed as

$$\begin{array}{c} \begin{array}{c} li_{dq0}=Pi_{abc}\\ v_{dq0}=Pv_{abc}\\ \psi_{dq0}=P\psi_{abc} \end{array} \end{array}$$ (17)

and their inverse transformations are

$$\begin{array}{c} \begin{array}{cc} i_{abc}= & P^{-1}i_{dq0}\\ v_{abc}= & P^{-1}v_{dq0}\\ \psi_{abc}= & P^{-1}\psi_{dq0} \end{array} \end{array}$$ (18)

The conventions of the source or generator are used to write Kirchhoff’s voltage-law equations for the *a*, *b*, and *c* stator windings. By applying Kirchhoff’s voltage law, the following equations are obtained

$$\begin{array}{c} v_{k}=R_{k}i_{k}+\frac{d\psi_{k}}{dt} \end{array}$$ (19)

where *k* = *a*, *b*, and *c*. Combining these equations gives

$$\begin{array}{c} P^{-1}v_{dq0}=R_{a}P^{-1}i_{dq0}+\frac{d(P^{-1}\psi_{dq0})}{dt} \end{array}$$ (20)

Pre-multiplying both sides of the equation by *P* results in

$$\begin{array}{c} v_{dq0}=R_{a}i_{dq0}+P\frac{d(P^{-1}\psi_{dq0})}{dt} \end{array}$$ (21)

After simplification, the equations for the q-axis and d-axis voltages become

$$\begin{array}{c} \begin{array}{c} v_{d}=R_{a}i_{d}+\frac{d\psi_{d}}{dt}-\psi_{q}\\ v_{q}=R_{a}i_{q}+\frac{d\psi_{q}}{dt}+\psi_{d} \end{array} \end{array}$$ (22)

The fluxes *ψ*_*d*_ and *ψ*_*q*_ produced in the direct and quadrature axis windings, respectively, are defined as

$$\begin{array}{c} \begin{array}{cc} \psi_{d}= & L_{d}i_{d}+\varphi_{m}\\ \psi_{q}= & L_{q}i_{q} \end{array} \end{array}$$ (23)

After simplification, the final *dq*-model equations for the PMSG are

$$\begin{array}{c} \begin{array}{cc} v_{d}= & R_{a}i_{d}+L_{d}\frac{d}{dt}i_{d}-L_{q}i_{q}\\ v_{q}= & R_{a}i_{q}+L_{q}\frac{d}{dt}i_{q}+\left(L_{d}i_{d}+\varphi_{m}\right) \end{array}\} \end{array}$$ (24)

where *v*_*q*_ and *v*_*d*_ are the *dq*-axis voltages, *L*_*q*_ and *L*_*d*_ are the rotor inductances, *ω*_*m*_ is the permanent magnet flux, and *R*_*a*_ is stator resistance. *L*_*d*_ = *L*_*q*_ = *L* for a non-salient PMSG.

### 2.4 State-space representation of system dynamics

Our system comprises of three states: *i*_*d*_, *i*_*q*_, and *ω*_*h*_ =. To simplify the system state equations for controller design, we express the equations of the PMSG in the form of nonlinear dynamical equations as follows

$$\begin{array}{c} \begin{array}{cc} {\dot{x}}_{1}= & \frac{-R_{s}x_{1}+p\left(L_{q}-L_{ch}\right)x_{2}x_{3}-R_{ini}x_{1}}{\left(L_{d}+L_{ch}\right)}\\ {\dot{x}}_{2}= & \frac{-R_{s}x_{2}-p\left(L_{d}+L_{ch}\right)x_{1}x_{3}-R_{ini}x_{2}}{\left(L_{q}+L_{ch}\right)}+p\varphi_{m}x_{3}\\ {\dot{x}}_{3}= & \frac{\frac{d_{1}{v_{w}}^{2}}{i}+\frac{d_{2}v_{w}x_{3}}{i^{3}}+\frac{d_{3}{x_{3}}^{2}}{i^{3}}-p\varphi_{m}x_{2}}{J_{h}} \end{array}\} \end{array}$$ (25)

where *x*_1_, *x*_2_, and *x*_3_ are the states *i*_*d*_, *i*_*q*_, and *ω*_*h*_, respectively; *p* is the pole pair number; *L*_*q*_, *L*_*d*_ and *L*_*ch*_ are the quadrature-axis, direct-axis, and chopper inductances, respectively; *R*_*ini*_ and *R*_*s*_ represent the chopper equivalence and stator resistances, respectively; *φ*_*m*_ denotes the flux; *d*_1_, *d*_2_, and *d*_3_ are constants; *v*_*w*_ is the wind velocity; and *J*_*h*_ represents the moment of inertia.

The state equations for the system (25) can also be written as

$$\begin{array}{c} \begin{array}{cc} \left[\begin{array}{c} {\dot{x}}_{1}\\ {\dot{x}}_{2}\\ {\dot{x}}_{3} \end{array}\right] & =[\begin{array}{c} \frac{-R_{s}x_{1}+p\left(L_{q}-L_{ch}\right)x_{2}x_{3}}{\left(L_{d}+L_{ch}\right)}\\ \frac{-R_{s}x_{2}-p\left(L_{d}+L_{ch}\right)x_{1}x_{3}}{\left(L_{q}+L_{ch}\right)}+p\varphi_{m}x_{3}\\ \frac{\frac{d_{1}{v_{w}}^{2}}{i}+\frac{d_{2}v_{w}x_{3}}{i^{3}}+\frac{d_{3}{x_{3}}^{2}}{i^{3}}-p\varphi_{m}x_{2}}{J_{h}} \end{array}]+[\begin{array}{c} \frac{-x_{1}}{\left(L_{d}+L_{ch}\right)}\\ \frac{-x_{2}}{\left(L_{q}+L_{ch}\right)}\\ 0 \end{array}]u\\ y & =h\left(x\right)=[\begin{array}{ccc} 0 & 0 & 1 \end{array}][\begin{array}{c} x_{1}\\ x_{2}\\ x_{3} \end{array}] \end{array} \end{array}$$ (26)

The parameters of the PMSG used in the system (26) are listed in Table 2.

**Table 2:** Parameters of the PMSG.

Parameters	Symbol	Value	Units
Stator Resistance	*R*_*s*_	3.3	Ohm
Direct-axis Inductance	*L*_*d*_	41.56 × 10^−3^	H
Quadrature-axis Inductance	*L*_*q*_	41.56 × 10^−3^	H
Chopper Inductance	*L*_*ch*_	0.08	Henry
Pole Pair Number	*p*	3	-
Flux	*φ*_*m*_	0.4382	Wb
Chopper Equivalence Resistance	*R*_*ini*_	80	Ohm

### 2.5 Relative degree of the system

Before moving on to the next subsection, which is the input-output form transformation, we need to determine the relative degree of the system to ensure that it meets the requirements for controller design. The relative degree, denoted as *r*, of the PMSG-WECS, can be determined by satisfying the following condition

$$\begin{array}{c} L_{g}L_{f}^{r-1}h\left(x\right)\neq 0\;\;\;\;\;\;\text{For}\;\text{values}\;\text{of}\;\;r=1,2,3,\ldots \end{array}$$ (27)

where *L*_*g*_ and *L*_*f*_ represent the Lie derivatives with respect to vector fields *g*(*x*) and *f*(*x*), respectively. According to the nonlinear system representation, the nonlinear functions *f*(*x*) and *g*(*x*) can be obtained from Eq (26) as follow

$$\begin{array}{c} \begin{array}{c} f\left(x\right)=[\begin{array}{c} f_{1}\left(x\right)\\ f_{2}\left(x\right)\\ f_{3}\left(x\right) \end{array}]=[\begin{array}{c} \frac{-R_{s}x_{1}+p\left(L_{q}-L_{ch}\right)x_{2}x_{3}}{\left(L_{d}+L_{ch}\right)}\\ \frac{-R_{s}x_{2}-p\left(L_{d}+L_{ch}\right)x_{1}x_{3}}{\left(L_{q}+L_{ch}\right)}+p\varphi_{m}x_{3}\\ \frac{1}{J_{h}}(\frac{d_{1}{v_{w}}^{2}}{i}+\frac{d_{2}v_{w}x_{3}}{i^{3}}+\frac{d_{3}{x_{3}}^{2}}{i^{3}}-p\varphi_{m}x_{2}) \end{array}]\\ g\left(x\right)=[\begin{array}{c} g_{1}\left(x\right)\\ g_{2}\left(x\right)\\ g_{3}\left(x\right) \end{array}]=[\begin{array}{c} \frac{-x_{1}}{\left(L_{d}+L_{ch}\right)}\\ \frac{-x_{2}}{\left(L_{q}+L_{ch}\right)}\\ 0 \end{array}],\;\;\;u=R_{ini} \end{array} \end{array}$$ (28)

To find the relative degree, we assume *r* = 1, and then the Lie derivative of *h* in the direction of *g* can be calculated as follows

$$\begin{array}{c} L_{g}L_{f}^{r-1}h\left(x\right)=L_{g}h\left(x\right)=\frac{\partial h(x)}{\partial (x)}\cdot g\left(x\right)=[\begin{array}{ccc} 0 & 0 & 1 \end{array}][\begin{array}{c} \frac{-x_{1}}{\left(L_{d}+L_{ch}\right)}\\ \frac{-x_{2}}{\left(L_{q}+L_{ch}\right)}\\ 0 \end{array}]=0 \end{array}$$ (29)

Because the relative degree *r* = 1 of the system does not satisfy this condition, we proceed to check for *r* = 2.

$$\begin{array}{c} L_{g}L_{f}h\left(x\right)=\frac{p\varphi_{m}x_{2}}{J_{h}\left(L_{q}+L_{ch}\right)}\neq 0 \end{array}$$ (30)

Thus, the relative degree of the given PMSG-based WECS is 2.

The following subsection transforms the system into a convenient input-output control form.

### 2.6 Input-output form transformation

The mathematical model of a generalized nonlinear system with input $u\in R^{m}$, output $y=h\left(x\right)\in R^{l}$, and state vector $x\in R^{n}$ is expressed as

$$\begin{array}{c} \begin{array}{cc} \dot{x} & =f(x)+g(x)u\\ y & =h(x) \end{array} \end{array}$$ (31)

where $f\left(x\right)\in R^{n}$ represents the nonlinear smooth state vector field and $g\left(x\right)\in R^{n\times m}$ is a matrix of smooth functions. In the given PMSG-WECS system, the state vector *x* is defined as

$$\begin{array}{c} x=[\begin{array}{c} x_{1}\\ x_{2}\\ x_{3} \end{array}]=[\begin{array}{c} i_{d}\\ i_{q}\\ \omega_{h} \end{array}] \end{array}$$ (32)

The dynamics of the system can be written as

$$\begin{array}{c} \begin{array}{cc} {\dot{x}}_{1} & =-k_{1}x_{1}-k_{2}x_{2}x_{3}-k_{3}ux_{1}\\ {\dot{x}}_{2} & =-l_{1}x_{2}-l_{2}x_{1}x_{3}+l_{3}x_{3}-l_{4}ux_{2}\\ {\dot{x}}_{3} & =-m_{1}-m_{2}x_{3}-m_{3}x_{3}^{2}-m_{4}x_{2} \end{array} \end{array}$$ (33)

where the terms *f*(*x*) and *g*(*x*) are expressed as

$$\begin{array}{c} \begin{array}{cc} f(x)= & \left[\begin{array}{c} f_{1}\left(x\right)\\ f_{2}\left(x\right)\\ f_{3}\left(x\right) \end{array}]=[\begin{array}{c} -k_{1}x_{1}-k_{2}x_{2}x_{3}\\ -l_{1}x_{2}-l_{2}x_{1}x_{3}+l_{3}x_{3}\\ -m_{1}-m_{2}x_{3}-m_{3}x_{3}^{2}-m_{4}x_{2} \end{array}\right]\\ g(x)= & \left[\begin{array}{c} g_{1}\left(x\right)\\ g_{2}\left(x\right)\\ g_{3}\left(x\right) \end{array}]=[\begin{array}{c} -k_{3}x_{1}\\ -l_{4}x_{2}\\ 0 \end{array}\right] \end{array} \end{array}$$ (34)

Substituting Eqs (34) into (31), we obtain the following equations

$$\begin{array}{c} \begin{array}{cc} \left[\begin{array}{c} {\dot{x}}_{1}\\ {\dot{x}}_{2}\\ {\dot{x}}_{3} \end{array}\right] & =[\begin{array}{c} -k_{1}x_{1}-k_{2}x_{2}x_{3}\\ -l_{1}x_{2}-l_{2}x_{1}x_{3}+l_{3}x_{3}\\ -m_{1}-m_{2}x_{3}-m_{3}x_{3}^{2}-m_{4}x_{2} \end{array}]+[\begin{array}{c} -k_{3}x_{1}\\ -l_{4}x_{2}\\ 0 \end{array}]u\\ y & =h\left(x\right)=[\begin{array}{ccc} 0 & 0 & 1 \end{array}][\begin{array}{c} x_{1}\\ x_{2}\\ x_{3} \end{array}] \end{array} \end{array}$$ (35)

In Eq (35), the control input *u* is given by

$$\begin{array}{c} u=R_{ini} \end{array}$$ (36)

The intermediate variables *z* in the inverse coordinate transformation are defined as

$$\begin{array}{c} z=[z_{1},L_{f}h\left(x\right),L_{f}^{2}h\left(x\right),\ldots ,L_{f}^{r-1}h\left(x\right)] \end{array}$$ (37)

The terms *z*_1_, *z*_2_, and *z*_3_ are expressed as follows

$$\begin{array}{c} \begin{array}{cc} z_{1} & =x_{3}\\ z_{2} & =L_{f}h\left(x\right)=[\begin{array}{ccc} 0 & 0 & 1 \end{array}][\begin{array}{c} -k_{1}x_{1}-k_{2}x_{2}x_{3}\\ -l_{1}x_{2}-l_{2}x_{1}x_{3}+l_{3}x_{3}\\ -m_{1}-m_{2}x_{3}-m_{3}x_{3}^{2}-m_{4}x_{2} \end{array}]\\ z_{3} & =L_{f}^{2}h\left(x\right)=\frac{x_{1}}{x_{2}} \end{array} \end{array}$$ (38)

The inverse coordinate transformation is given by

$$\begin{array}{c} \begin{array}{cc} x_{1} & =z_{3}\frac{\left(m_{1}-z_{2}-m_{2}z_{1}-m_{3}z_{1}^{2}\right)}{m_{4}}\\ x_{2} & =\frac{\left(m_{1}-z_{2}-m_{2}z_{1}-m_{3}z_{1}^{2}\right)}{m_{4}}\\ x_{3} & =z_{1} \end{array} \end{array}$$ (39)

Let us now examine system dynamics in the z-domain. The equations can be written as follows

$$\begin{array}{c} \begin{array}{cc} {\dot{z}}_{1}= & {\dot{x}}_{3}=z_{2}\\ {\dot{z}}_{2}= & L_{f}^{2}h\left(x\right)+L_{g}L_{f}h\left(x\right)u \end{array} \end{array}$$ (40)

The Lie derivatives are expressed as

$$\begin{array}{c} \begin{array}{cc} L_{f}^{2}h\left(x\right) & =-m_{4}f_{2}-\left(m_{2}+2m_{3}x_{3}\right)f_{3}\\ L_{g}L_{f}h\left(x\right) & =l_{4}m_{4}x_{2} \end{array} \end{array}$$ (41)

Therefore, the linearized model of the system is

$$\begin{array}{c} \begin{array}{cc} {}[\begin{array}{c} {\dot{z}}_{1}\\ {\dot{z}}_{2} \end{array}]= & [\begin{array}{cc} 0 & 1\\ 0 & 0 \end{array}][\begin{array}{c} z_{1}\\ z_{2} \end{array}]+[\begin{array}{c} 0\\ 1 \end{array}]u\\ y= & \left[\begin{array}{cc} 1 & 0 \end{array}][\begin{array}{c} z_{1}\\ z_{2} \end{array}\right] \end{array} \end{array}$$ (42)

The control input *u* will be designed using the “Arbitrary order based sliding mode controller”.

In the next subsection, we investigate whether the third internal dynamic state of the system is stable or not.

### 2.7 Zero-dynamic stability investigation of the system

After all the calculations and transformation into a z-coordinate system, we obtained the third internal dynamic state of the system, which is given by

$$\begin{array}{c} \begin{array}{cc} {\dot{z}}_{3} & =\frac{m_{4}}{m_{1}}(-\frac{k_{1}z_{3}m_{1}}{m_{4}}-\frac{k_{2}z_{1}m_{1}}{m_{4}}-\frac{k_{3}z_{3}m_{1}u}{m_{4}})\\ & \;-\frac{z_{3}m_{1}}{m_{4}}(\frac{m_{4}^{2}}{m_{1}^{2}})(-\frac{l_{1}m_{1}}{m_{4}}-\frac{l_{2}m_{1}z_{3}z_{1}}{m_{4}}+l_{3}z_{1}-\frac{l_{4}m_{1}u}{m_{4}}) \end{array} \end{array}$$ (43)

To prove the stability of the zero-dynamic state, the following variables must be assigned zero: *z*_1_ = *z*_2_ = *u* = 0. By simplifying Eq (43), we can derive the following expression

$$\begin{array}{c} {\dot{z}}_{3}=-z_{3}\left(k_{1}-l_{1}\right) \end{array}$$

Since the design constant *k*_1_ > *l*_1_, we have

$$\begin{array}{c} {\dot{z}}_{3}=-k_{1}z_{3} \end{array}$$ (44)

where the zero-dynamic state is stable for all *k*_1_ > *l*_1_.

In the next step, we will design a control algorithm to adjust the duty cycle and maximize power extraction from the wind turbine system.

## 3 Arbitrary order sliding mode control design

**Fig 5:**
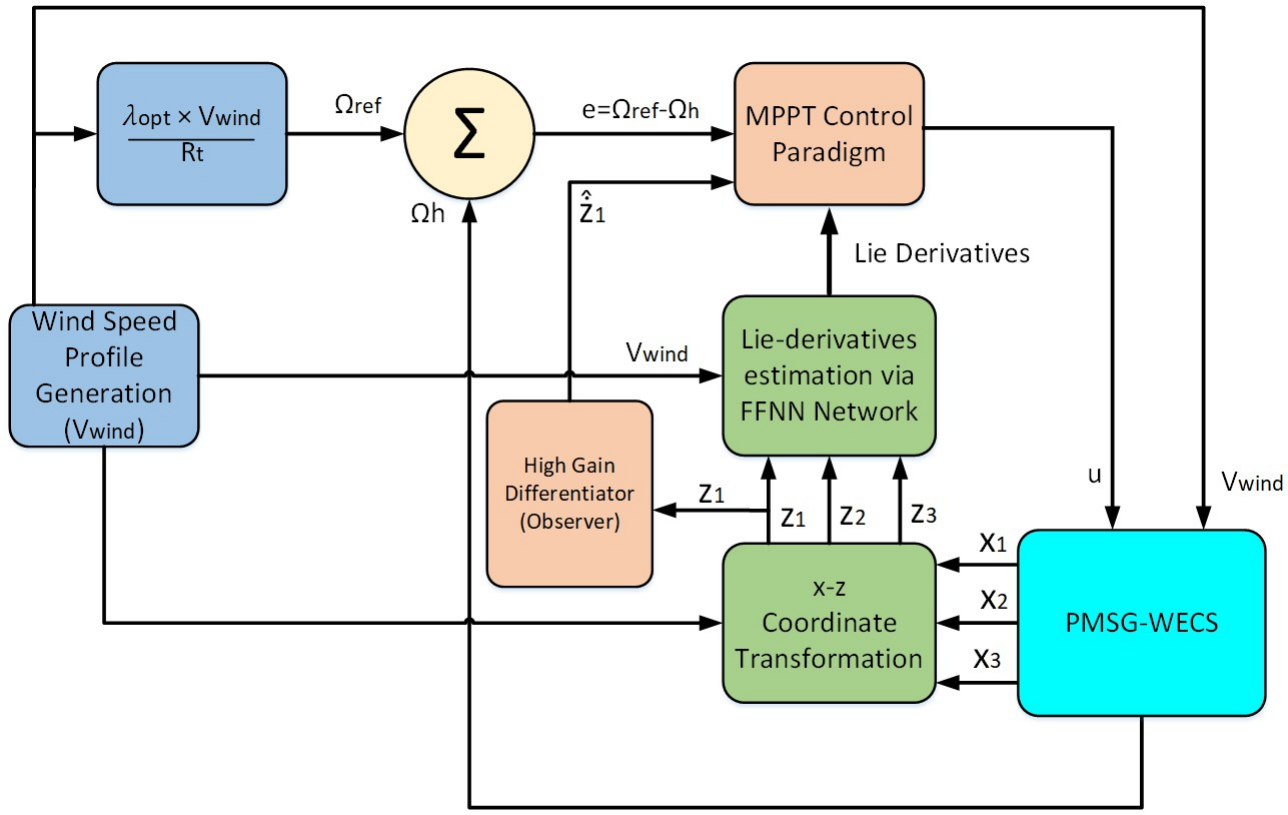
Block diagram of overall control system.

In this section, a neuro-adaptive arbitrary order sliding mode control technique is proposed for Maximum Power Extraction (MPE) while handling external disturbances and parametric uncertainties with high accuracy [44]. This technique introduces some nonlinear terms to the sliding manifold, enabling the establishment of a sliding mode in finite time. Moreover, the state convergence is independent of the initial conditions. The proposed law effectively reduces undesirable fluctuations that can impact the rotor model. Fig 5 shows the block diagram of the overall control system. To decrease the system sensitivity to uncertain disturbances, a neural network and differentiator-based AOSMC are designed to track the wind reference speed and consequently achieve the maximum power extraction from the wind turbine. For the control design of the proposed AOSMC, we refer to the output equations presented in (42) and (44) under the assumption that the internal (or zero dynamics) are stable.

The proposed neuroadaptive control design is described in the following subsection.

### 3.1 Feedforward neural network architecture

In this subsection, we implement the approximation of the input channel *L*_*g*_*L*_*f*_*h*(*x*) and the nonlinear drift term $L_{f}^{2}h\left(x\right)$ using a feedforward neural network. The specific approximation functions used depend on system parameters.

**Fig 6:**
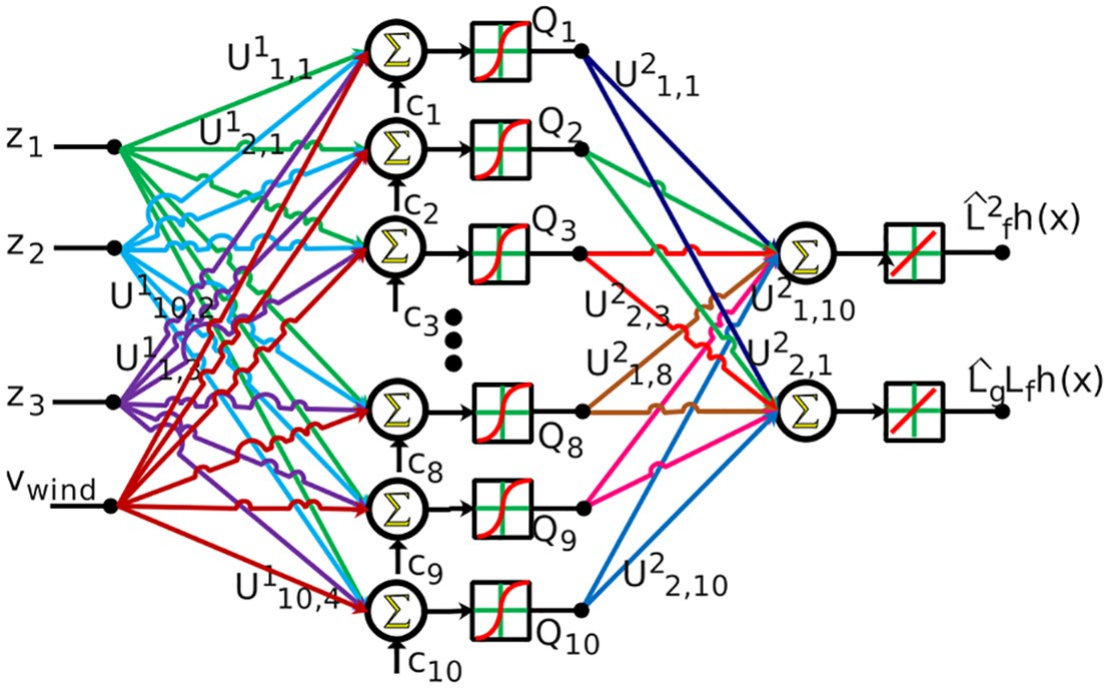
Neural network for the approximation of $L_{f}^{2}h\left(x\right)$ and *L*_*g*_*L*_*f*_*h*(*x*).

We employed a three-layer feedforward neural network consisting of an input layer, an output layer, and a hidden layer of *N* neurons [45]. Mapping of input data to output/target data to train the network. The network inputs are the z-transform states (*z*_1_, *z*_2_, *z*_3_) and the wind speed *V*_wind_, while the network output is the approximation of the targeted values (i.e., the input channel *L*_*g*_*L*_*f*_*h*(*x*) and the nonlinear drift term $L_{f}^{2}h\left(x\right)$). The z states are input to the network along with the wind speeds, as shown in Fig 6. The network outputs corresponding to the lie derivatives are utilized in the AOSMC design.

The FFNN can be represented as follows

$$\begin{array}{c} \begin{array}{cc} Q_{N} & =f_{N}^{1}(\sum_{n=1}^{N}U_{N,n}^{1}Y+c_{N}^{1})=f_{N}^{1}(U_{N}^{1^{T}}+c_{N}^{1})\\ {{\hat{L}}^{2}}_{f}h\left(x\right) & =f_{m}^{2}(\sum_{N=1}^{m}U_{{L^{2}}_{f}h\left(x\right),N}^{2}Q_{N})=U_{{L^{2}}_{f}h\left(x\right),N}^{2^{T}}Q_{N}\\ {\hat{L}}_{g}L_{f}h\left(x\right) & =f_{m}^{2}(\sum_{N=1}^{m}U_{L_{g}L_{f}h\left(x\right),N}^{2}Q_{N})=U_{L_{g}L_{f}h\left(x\right),N}^{2^{T}}Q_{N} \end{array} \end{array}$$ (45)

where *m* denotes the number of network outputs, *n* the number of network inputs, *M* the number of output layer neurons, and *N* the number of hidden layer neurons. The input vector $Y={\left[z_{1},z_{2},z_{3},V_{\text{wind}}\right]}^{T}\in R^{n}$ is fed into the network. The target outputs of the network, ${{\hat{L}}^{2}}_{f}h\left(x\right)$ and ${\hat{L}}_{g}L_{f}h\left(x\right)$, are obtained. The hidden layer output vector is denoted as $Q_{N}\in R^{N}$, while $U_{{L^{2}}_{f}h\left(x\right),N}^{2}\in R^{N}$ and $U_{L_{g}L_{f}h\left(x\right),N}^{2}\in R^{N}$ represent the weight vectors of output-layer. The bias term $c^{1}\in R^{N}$ enhances the learning speed during the training of network.

In the next section, we describe the design of a high-gain differentiator to approximate higher-order derivatives of the system.

### 3.2 State estimator design using high gain differentiator

This section introduces the High Gain Observer (HGO) as a state estimator. HGO estimates the higher derivatives of the system states, which are not directly measurable and are required in the controller as known data. The motivation for using HGO is twofold: first, it provides fast convergence to the actual values; second, it has been used in the existing literature, making it a suitable choice for our work.

Consider a general nonlinear system represented by the equations

$$\begin{array}{c} \begin{array}{cc} {\dot{\eta }}_{i}= & \eta_{i+1}\\ {\dot{\eta }}_{n}= & \vartheta (\eta ,z,t) \end{array} \end{array}$$ (46)

where *i* = 1, 2, …, *n* − 1, *η* denotes the measurable state vector, *z* represents the controlled input, and *ϑ*(*η*, *z*, *t*) represents a nonlinear (or possibly linear) function involving these variables. It is assumed that *ϑ*(*η*, *z*, *t*) is locally Lipschitz if there exists *L* ≥ 0 satisfying the condition

$$\begin{array}{c} |\vartheta (\eta ,z,t)-\vartheta (x,z,t)|\leq L\left|\eta -x\right| \end{array}$$

uniformly, with respect to *η* and *x*, where *x* denotes the observer state. The parameter *L* is typically dependent on the radii of the neighborhoods (refer to [46] for further information).

In our study, we assume that only *η*_1_ is accessible, whereas higher derivatives require estimation. To fulfil this requirement, we employ an observer of the following structure

$$\begin{array}{c} \begin{array}{cc} {\dot{x}}_{1}=x_{2}+ & \frac{\alpha_{1}}{\epsilon }\left(\eta_{1}-x_{1}\right)\\ {\dot{x}}_{2}=x_{3}+ & \frac{\alpha_{2}}{\epsilon^{2}}\left(\eta_{1}-x_{1}\right)\\ \vdots \\ {\dot{x}}_{n-1}=x_{n}+ & \frac{\alpha_{n-1}}{\epsilon^{n-1}}\left(\eta_{1}-x_{1}\right)\\ {\dot{x}}_{n}=\vartheta (x,v,t)+\frac{\alpha_{n}}{\epsilon^{n}}\left(\eta_{1}-x_{1}\right) \end{array} \end{array}$$ (47)

In a compact and abstract form, the observer can be expressed as [46]

$$\begin{array}{c} \dot{x}=E_{n}x+\alpha \left(\epsilon \right)\left(\eta_{1}-x_{1}\right)+D_{n}\vartheta \left(x,v,t\right) \end{array}$$ (48)

where $\alpha \left(\epsilon \right)=[\frac{\alpha_{1}}{\epsilon },\frac{\alpha_{2}}{\epsilon^{2}},\ldots ,\frac{\alpha_{n}}{\epsilon^{n}}]$ and *ϵ* is a bounded high gain, i.e., $0<\epsilon <\bar{\epsilon }$. In general, *ϵ* is chosen to be extremely small, approaching zero. As *ϵ* approaches zero, the gains of the injection terms in the observer become higher and higher, thus earning the term “high gain”. The matrices $E_{n}=[\begin{array}{cc} P_{(n-1)\times 1} & I_{\left(n-1)\times (n-1\right)}\\ P_{1\times 1} & P_{1\times (n-1)} \end{array}]$ and $D_{n}=[\begin{array}{c} P_{(n-1)\times 1}\\ I_{1\times 1} \end{array}]$ have special structures.

In the next section, we will design the proposed AOSM control technique for tracking the wind power system’s Maximum Power Point (MPP).

### 3.3 Control law design

Firstly, we define the tracking error as follows to fulfil the main objective of tracking the reference speed

$$\begin{array}{c} e=z_{1}-z_{\text{ref}} \end{array}$$ (49)

To determine the control input, the double time derivative of the error is

$$\begin{array}{c} \begin{array}{c} \dot{e}=z_{2}-{\dot{z}}_{\text{ref}}\\ \ddot{e}={\dot{z}}_{2}-{\ddot{z}}_{\text{ref}} \end{array} \end{array}$$ (50)

The novelty of this study lies in the sliding surface design, which provides the aforementioned benefits. Thus, the proposed sliding surface [44] is expressed as follows

$$\begin{array}{c} s=\dot{e}+\lambda e+\int_{0}^{t}Id\tau \end{array}$$ (51)

with

$$\begin{array}{c} I=c_{2}|\dot{e}{|}^{\alpha_{2}}\text{sign}\left(\dot{e}\right)+c_{1}{\left|e\right|}^{\alpha_{1}}\text{sign}\left(e\right)+b_{2}|\dot{e}{|}^{\beta_{2}}\text{sign}\left(\dot{e}\right)+b_{1}{\left|e\right|}^{\beta_{1}}\text{sign}\left(e\right) \end{array}$$ (52)

where *α*_2_, *α*_1_, *β*_2_, *β*_1_, *c*_2_, *c*_1_, *b*_2_, and *b*_1_ are positive design constants. Taking the derivative of the sliding surface Eq (51), one can get

$$\begin{array}{c} \begin{array}{cc} & \dot{s}=\ddot{e}+\lambda \dot{e}+I\\ & \dot{s}={\dot{z}}_{2}-{\ddot{z}}_{\text{ref}}+\lambda \left(z_{2}-{\dot{z}}_{\text{ref}}\right)+I\\ & \dot{s}=L_{f}^{2}h\left(x\right)+L_{g}L_{f}h\left(x\right)u+\Delta -{\ddot{z}}_{\text{ref}}+\lambda \left(z_{2}-{\dot{z}}_{\text{ref}}\right)+I \end{array} \end{array}$$ (53)

After performing the necessary calculations and assuming $\dot{s}=0$ in Eq (53), the equivalent controller *u*_equ_ is as follows

$$\begin{array}{c} u_{\text{equ}}=\frac{1}{L_{g}L_{f}h\left(x\right)}(-L_{f}^{2}h\left(x\right)+{\ddot{z}}_{d}-\lambda \dot{e}-I) \end{array}$$ (54)

The conventional reachability term is given by

$$\begin{array}{c} u_{\text{dis}}=-k_{1}s-k_{2}\text{sign}\left(s\right) \end{array}$$ (55)

The overall controller equation is

$$\begin{array}{c} u=u_{\text{equ}}+u_{\text{dis}} \end{array}$$ (56)

Thus, the final design of the controller is as follows

$$\begin{array}{c} u=\frac{1}{L_{g}L_{f}h\left(x\right)}(-L_{f}^{2}h\left(x\right)+{\ddot{z}}_{d}-\lambda \dot{e}-I)-k_{1}s-k_{2}\text{sign}\left(s\right) \end{array}$$ (57)

where $I=c_{2}|\dot{e}{|}^{\alpha_{2}}\text{sign}\left(\dot{e}\right)+c_{1}{\left|e\right|}^{\alpha_{1}}\text{sign}\left(e\right)+b_{2}|\dot{e}{|}^{\beta_{2}}\text{sign}\left(\dot{e}\right)+b_{1}{\left|e\right|}^{\beta_{1}}\text{sign}\left(e\right)$.

### 3.4 Stability analysis

The main objective is to demonstrate the stability of the PMSG-WECS under Neuro-adaptive AOSMC. To achieve this, we utilized Lyapunov-based stability analysis. We define the Lyapunov function, which will be used to prove the enforcement of the sliding mode, as

$$\begin{array}{c} V=\frac{1}{2}s^{2} \end{array}$$ (58)

Taking the time derivative of *V*, we have

$$\begin{array}{c} \begin{array}{cc} & \dot{V}=s\dot{s}\\ & \dot{V}=-L_{g}L_{f}h\left(x\right)k_{1}s^{2}-L_{g}L_{f}h\left(x\right)k_{2}\text{sign}\left(s\right)s+\Delta s \end{array} \end{array}$$ (59)

To verify whether the Lyapunov function satisfies the condition of finite and bounded states, the identity sign(*s*)*s* = |*s*| is used. Therefore, Eq (59) becomes

$$\begin{array}{c} \begin{array}{cc} & \dot{V}\leq -L_{g}L_{f}h\left(x\right)k_{1}s^{2}-L_{g}L_{f}h\left(x\right)k_{2}\left|s\right|+\left|\Delta ||s\right|\\ & \dot{V}\leq -L_{g}L_{f}h\left(x\right)k_{1}s^{2}-|s|(L_{g}L_{f}h\left(x\right)k_{2}-\left|\Delta |\right) \end{array} \end{array}$$ (60)

We introduce the uncertainty term *η* into the above equation as

$$\begin{array}{c} L_{g}L_{f}h\left(x\right)k_{2}-\left|\Delta \right|\geq \eta \end{array}$$ (61)

Now, Eq (60) can be rewritten as

$$\begin{array}{c} \dot{V}\leq -L_{g}L_{f}h\left(x\right)k_{1}s^{2}-\left|s\right|\eta \end{array}$$ (62)

By substituting $\left|s\right|=\sqrt{2}V^{\frac{1}{2}}$ in Eq (62), we get

$$\begin{array}{c} \begin{array}{cc} & \dot{V}\leq -L_{g}L_{f}h\left(x\right)k_{1}2V-\eta \sqrt{2}V^{\frac{1}{2}}\\ & \dot{V}+L_{g}L_{f}h\left(x\right)k_{1}2V+\eta \sqrt{2}V^{\frac{1}{2}}\leq 0\\ & \dot{V}+\xi_{1}V+\xi_{2}V^{\frac{1}{2}}\leq 0 \end{array} \end{array}$$ (63)

where *ξ*_1_ = 2*L*_*g*_*L*_*f*_*h*(*x*)*k*_1_ and $\xi_{2}=\eta \sqrt{2}$. The above inequality clearly illustrates the achievement of rapid finite-time convergence as *V* approaches zero, indicating the simultaneous approach of both *s* and sliding mode to zero. To elaborate further, the determination of the finite-time upper bound depends on specific control parameters, system dynamics, and the desired convergence time, as expressed follows

$$\begin{array}{c} T_{f}\leq \frac{1}{2\xi_{1}}\text{ln}(\frac{\xi_{1}\sqrt{V}\left(s\left(0\right)\right)+\xi_{2}}{\xi_{2}}) \end{array}$$ (64)

The primary advantage of structuring the sliding mode surface with finite-time convergence, as presented in this research, lies in its capacity to achieve swifter and more predictable convergence compared to traditional SMC methods. Traditional SMC techniques often require more time to reach a stable state, which may not align with the demands of applications requiring rapid responses, such as those in wind energy systems.

## 4 Simulation results and discussion

This section discusses the simulation results of the proposed neuro-adaptive AOSMC algorithm for the maximum power extraction in a wind energy conversion system. Comparative investigations are conducted to evaluate the performance of the proposed technique in comparison to feedback linearization control (FBLC) [35] and generalized global sliding mode control (GGSMC) [36]. The simulations are performed on a standalone, fixed-pitch, variable-speed, 3 kW PMSG-based WECS. The wind profile consists of 100 seconds of data within the 2.0-10.4 m/s speed range, with an average speed of 7.0 m/s in Region 2.

In the following subsection, the figure illustrating the neural-network layers are presented, followed by the feedforward neural-network simulation results.

### 4.1 Simulation results of FFNN

**Fig 7:**
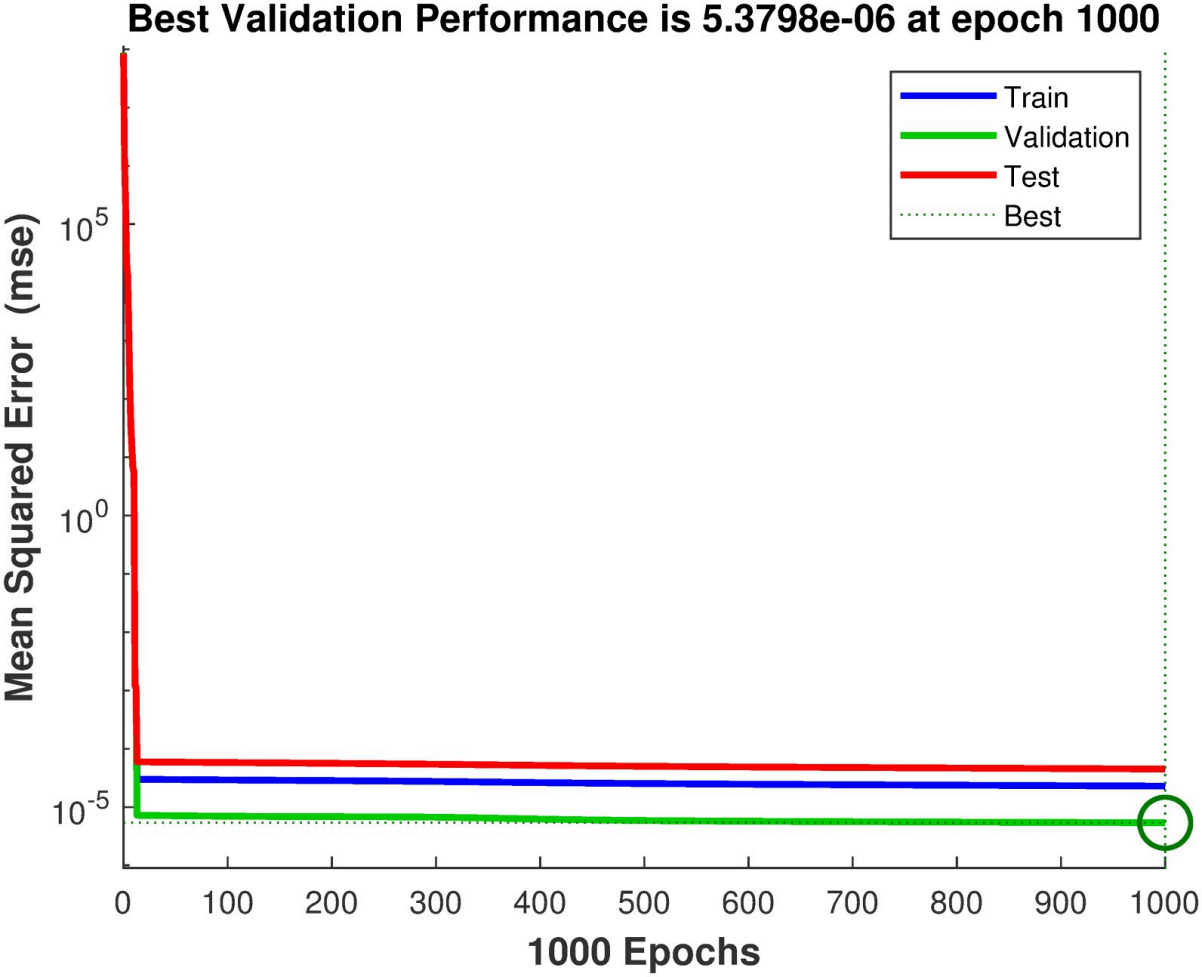
Performance function of feedforward back propagation neural network.

This subsection presents the simulation results of the feedforward neural network used to estimate the Lie derivatives $L_{f}^{2}h\left(x\right)$ and *L*_*g*_*L*_*f*_*h*(*x*). The performance in terms of mean squared error (MSE) during the estimation of $L_{f}^{2}h\left(x\right)$ and *L*_*g*_*L*_*f*_*h*(*x*) is evaluated and illustrated in Fig 7.

**Fig 8:**
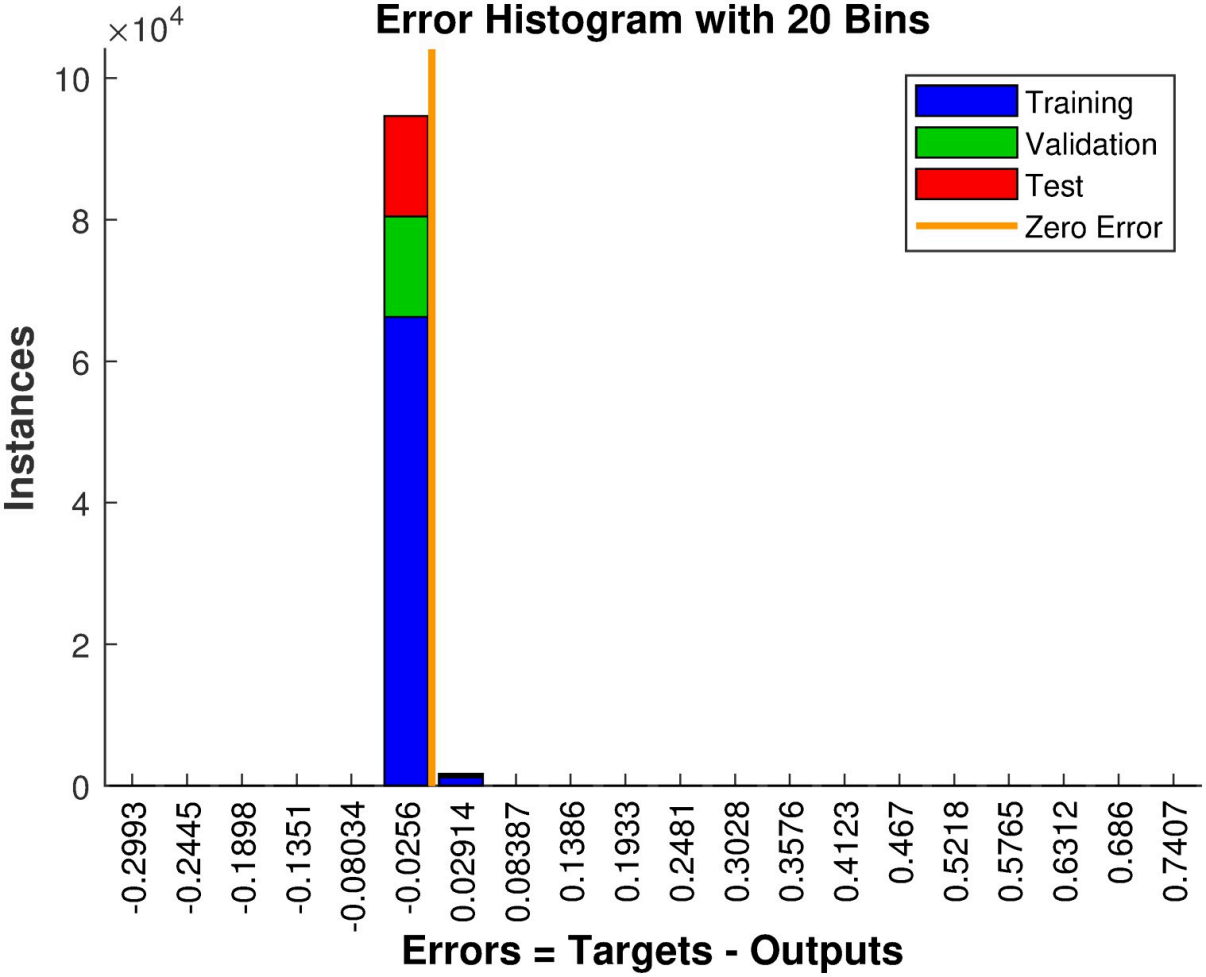
Error histogram.

Initially, there is a significant error; however, as the number of training epochs increased, the error gradually decreased. The estimation errors for $L_{f}^{2}h\left(x\right)$ and *L*_*g*_*L*_*f*_*h*(*x*) are analyzed using the error histogram shown in Fig 8.

**Fig 9:**
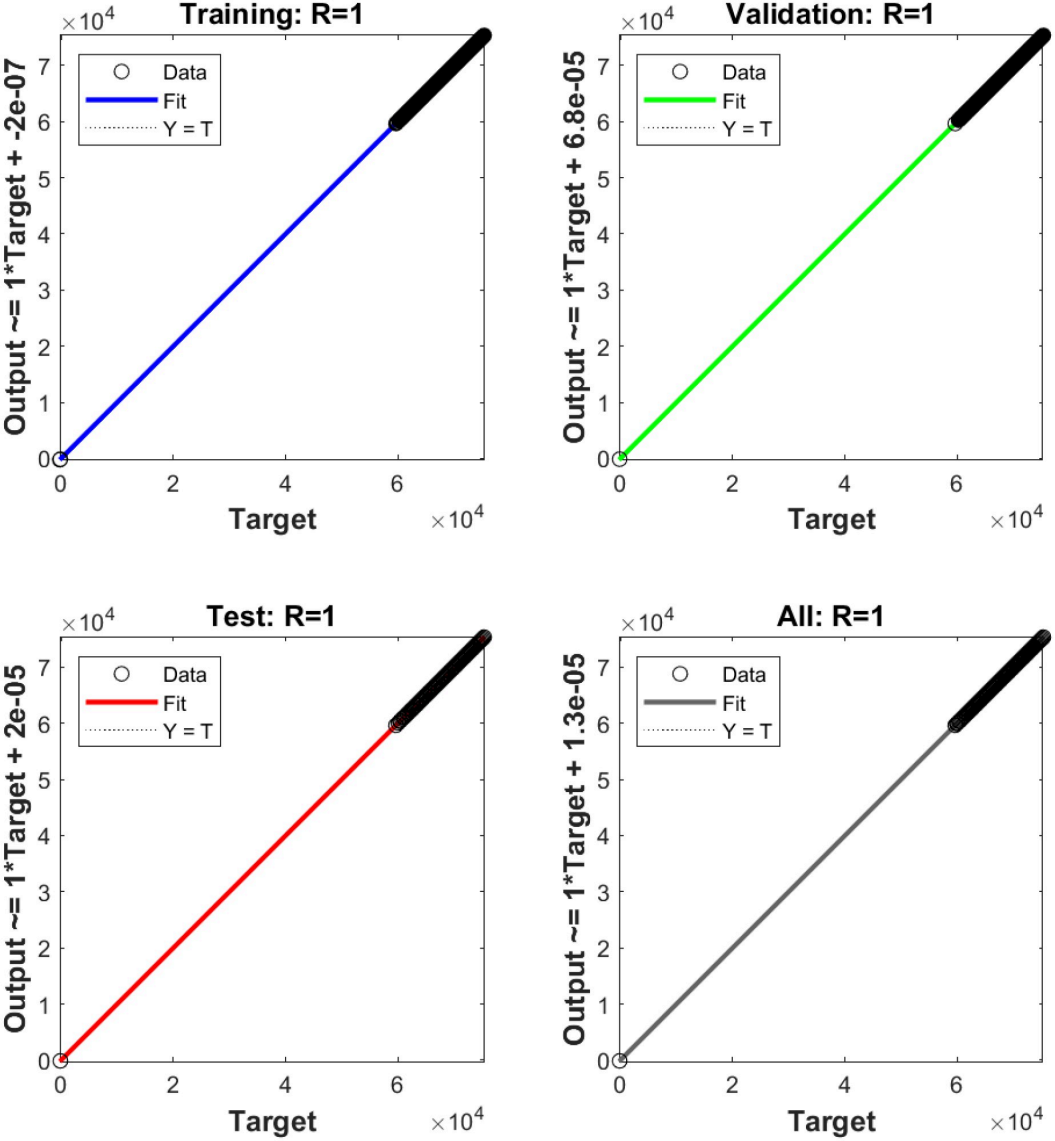
Regression plot of feedforward neural network.

The regression plots of the estimated values against the goal values are presented in Fig 9. The success rate of the estimation is determined using the regression parameter *R*. A value of *R* = 1 indicates reasonable estimates, whereas lower values of *R* signify lower estimation accuracy.

### 4.2 Simulation results of state estimator

**Fig 10:**
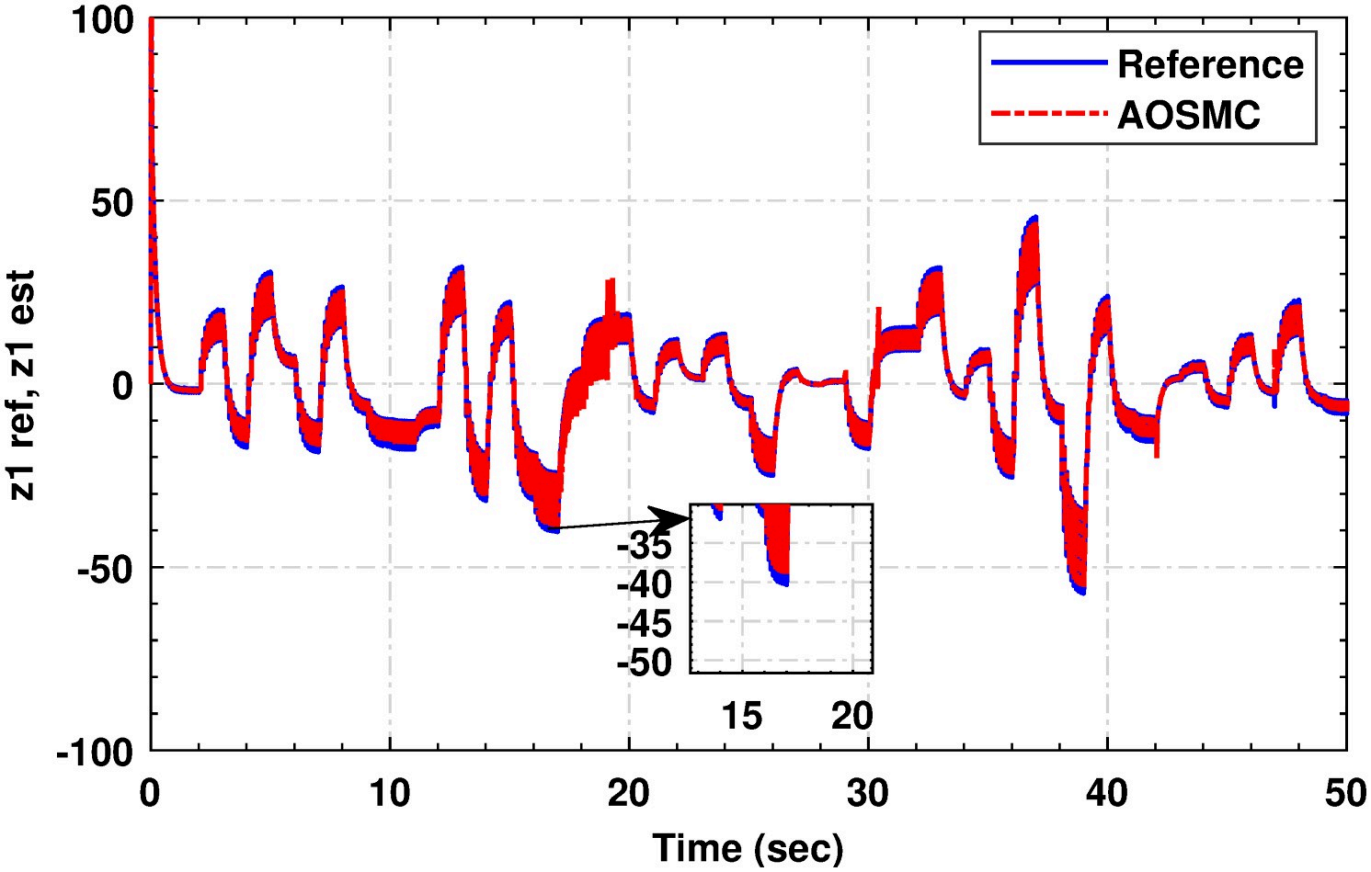
Actual and observed state.

The fast convergence of the proposed observer to zero ensures the stability of the closed-loop control system, as reflected in the boundedness of the high gain *ϵ* (see [46] and references related to HGO). It is worth noting that a high-gain observer is used throughout the simulation. The main distinction between the two is that the gain parameter *ϵ* in the high-gain observer does not always approach zero, unlike in the classical observer. The adoption of a high-gain observer is motivated by the need for asymptotic stability of the entire closed-loop system. The missing states are accurately calculated using the proposed differentiator, as illustrated in Fig 10.

In the following subsection, the results and comparisons between the AOSMC and GGSMC controllers are discussed in detail.

### 4.3 Result comparison of proposed controller

In this subsection, we compare the proposed AOSMC controller with the FBLC [35] and GGSMC [36] from the existing literature through graphical analyses. First, we generated the output of the AOSMC controller and compared it with that of GGSMC. The purpose of these comparisons is to highlight the superior performance of the proposed AOSMC law.

**Fig 11:**
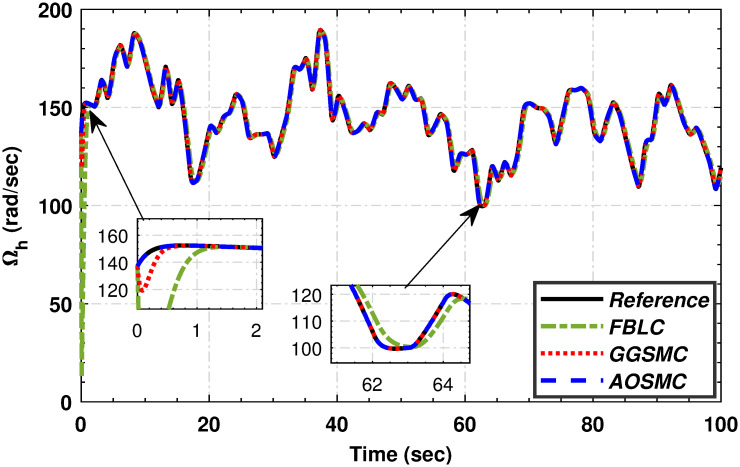
The tracking performance of high-speed shaft angular speed.

Fig 11 compares the PMSG wind–shaft speed tracking performance. The results indicate that the AOSMC outperforms the GGSMC, particularly in zoomed-in areas, demonstrating its superior wind-speed tracking capability. In addition, the AOSMC controller exhibited finite-time convergence, underscoring its effectiveness in achieving stable and accurate tracking.

**Fig 12:**
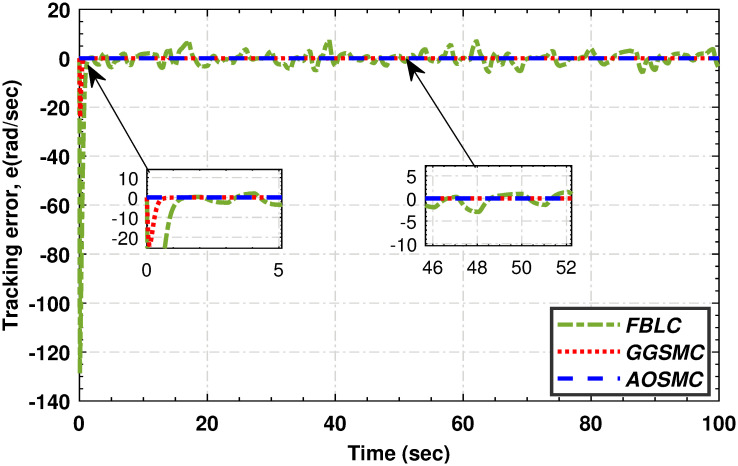
The tracking mismatch of high-speed shaft angular speed.

**Fig 13:**
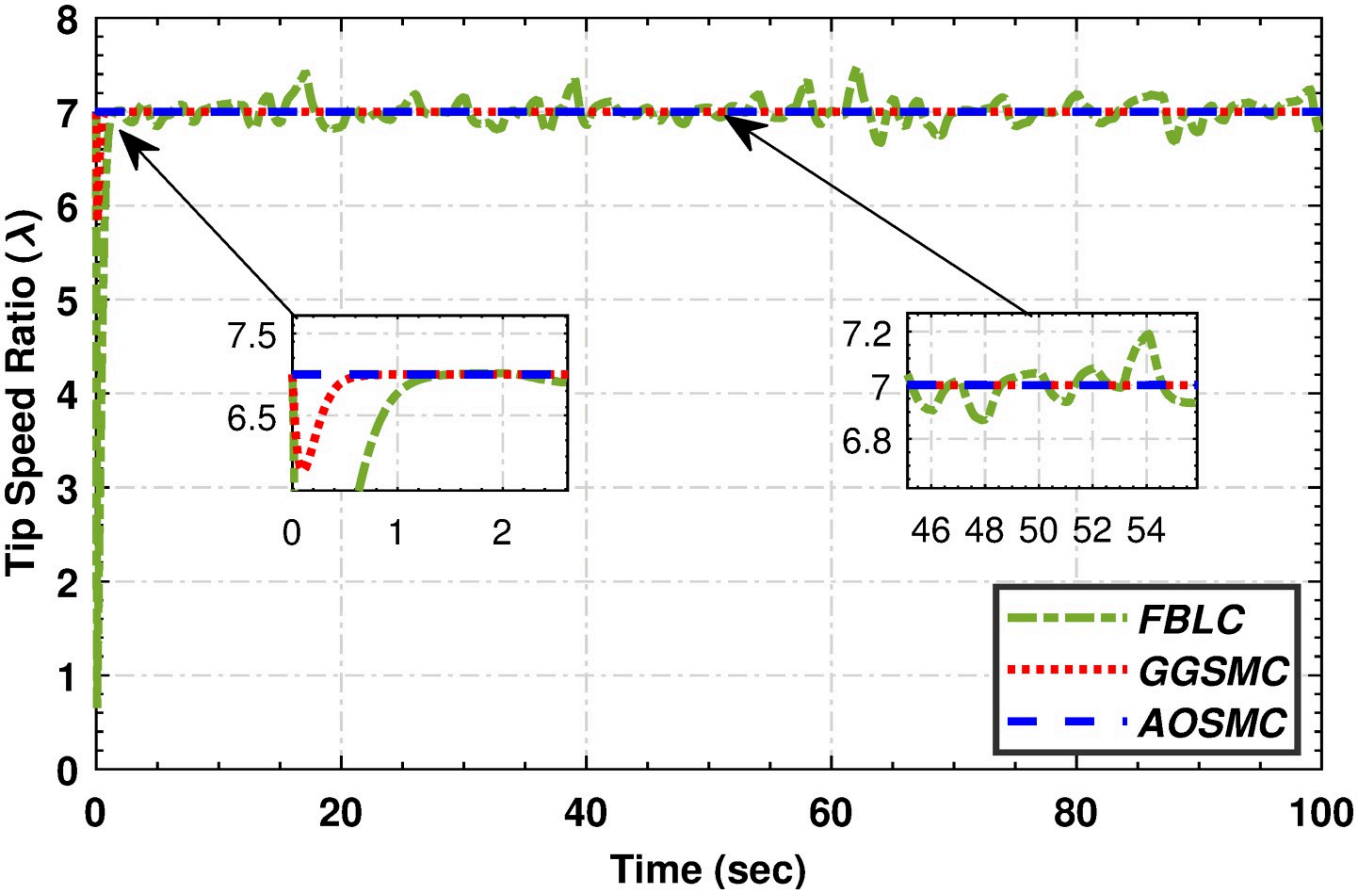
Tip speed ratio performance.

**Fig 14:**
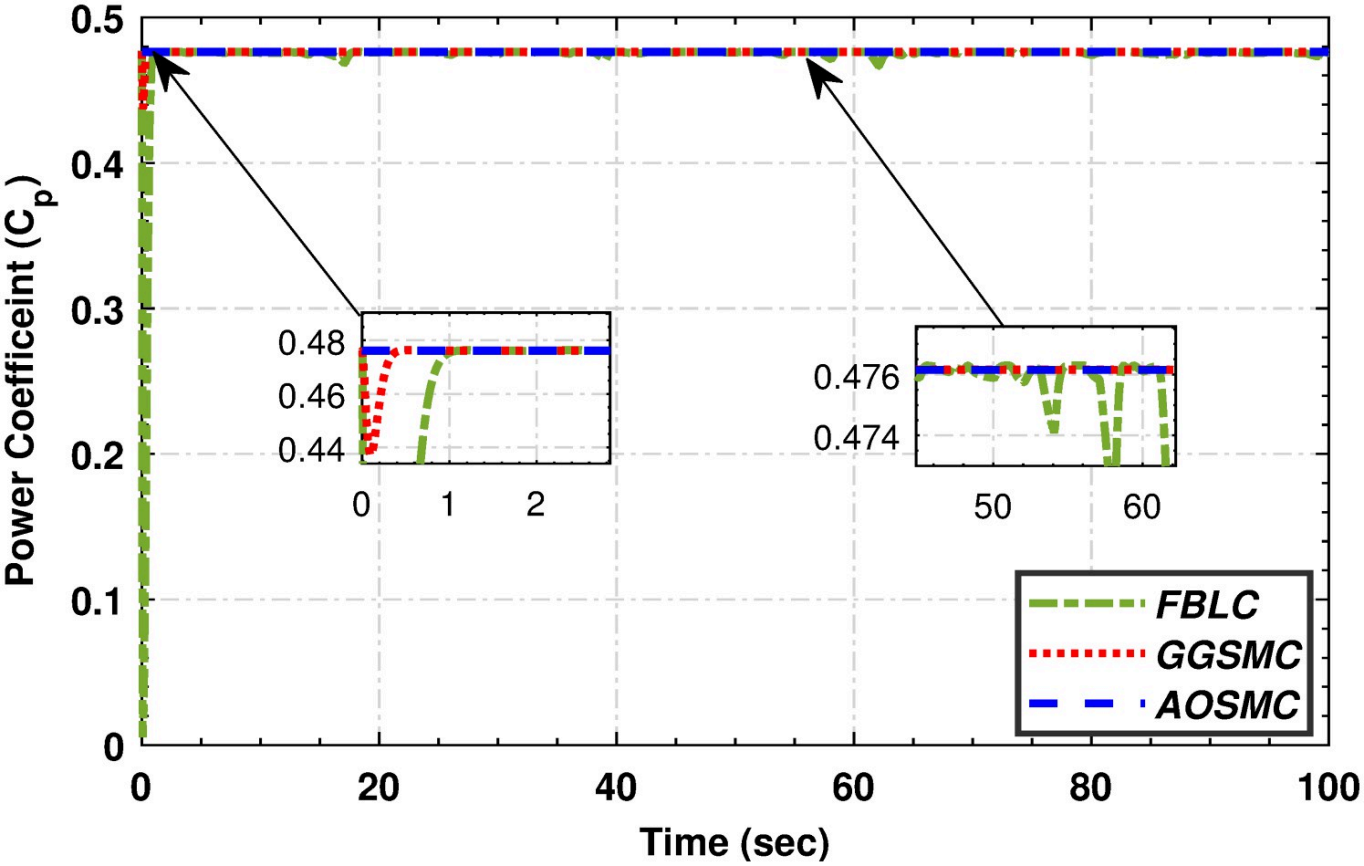
Power conversion coefficient.

When comparing the tracking performance of the PMSG wind shaft speed, Fig 12 reveals that the AOSMC controller surpasses the GGSMC controller in terms of accuracy, showcasing AOSMC’s robustness against initial fluctuations and its ability to achieve finite-time convergence. Additionally, the AOSMC controller exhibits exceptional precision in tracking the TSR of the variable-speed wind turbine (VSWT), as illustrated in Fig 13, with minimal oscillations around the optimal TSR (λ_*opt*_). This characteristic makes the AOSMC controller the preferred choice for MPPT. Furthermore, Fig 14 provides a comparative analysis of the power-conversion coefficients (*C*_*p*_) of the controllers. Throughout the specified wind speed profile, the AOSMC approach consistently tracked the optimal *C*_*p*_ value (*C*_*p*_ = 0.4762), thereby ensuring efficient power extraction. Specifically, under AOSMC, the zoomed-in areas of the aforementioned figures demonstrate minimal initial fluctuations, signifying its robustness against the uncertain disturbances. This robustness stems from AOSMC’s inherent ability of the AOSMC to rapidly counteract disturbances while maintaining oscillations to a minimum during the initial control actions. In contrast, GGSMC and FBLC may exhibit more pronounced fluctuations during these initial moments due to their susceptibility to disturbances and slower response times.

**Fig 15:**
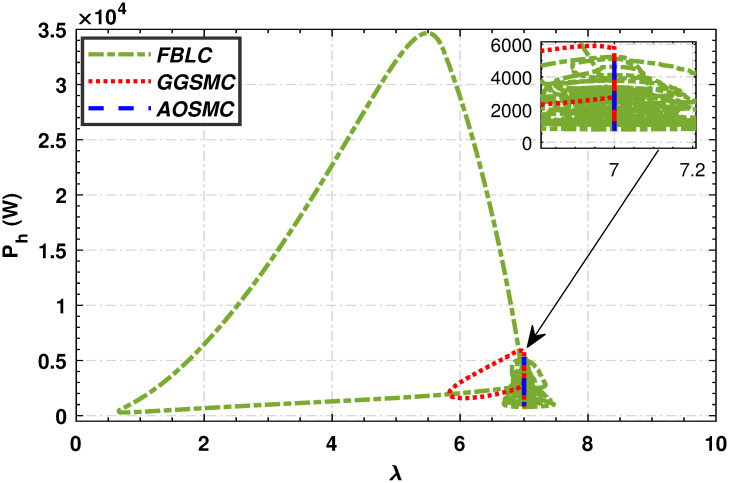
Mechanical power of the high-speed shaft in relation to the tip speed ratio.

**Fig 16:**
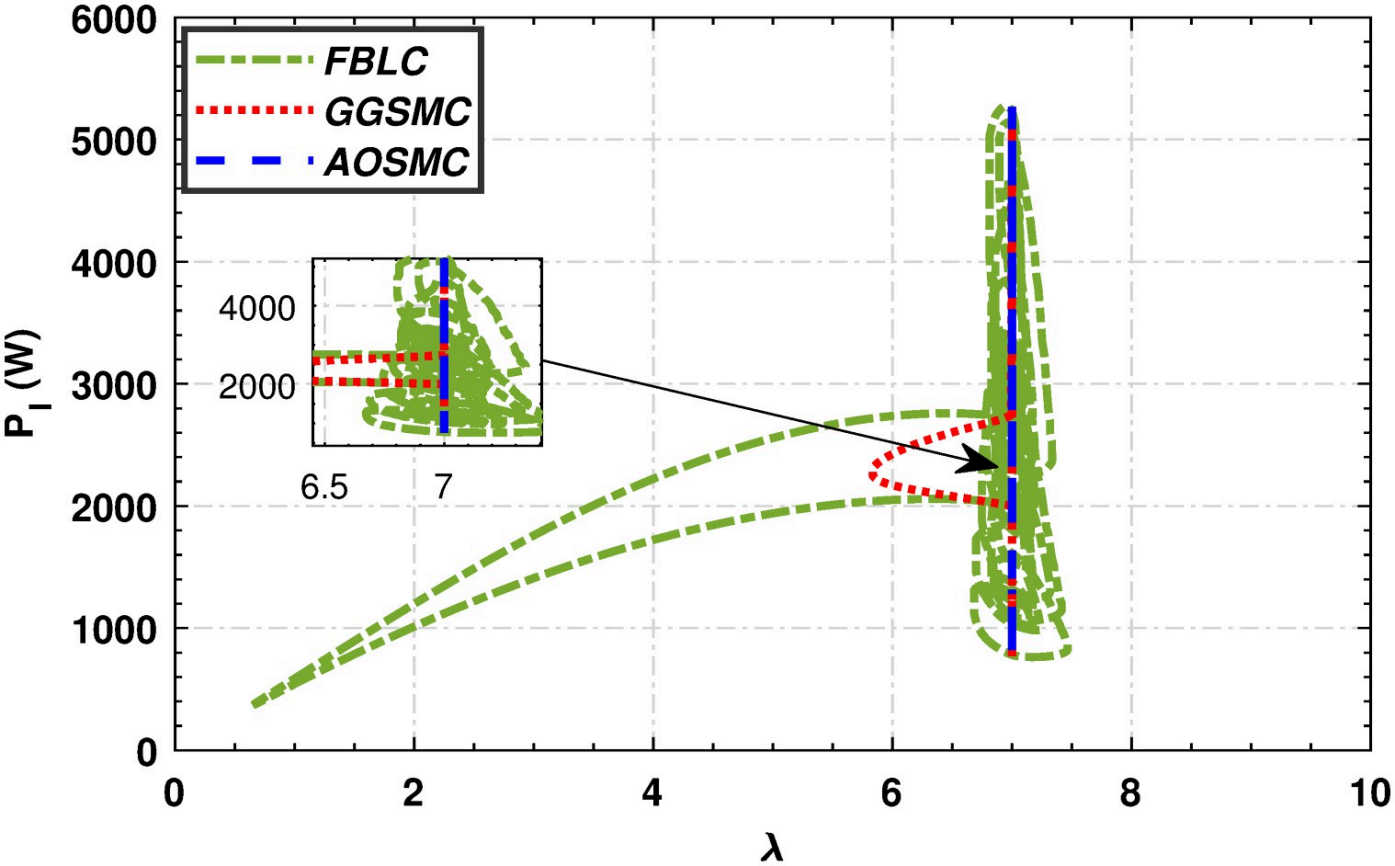
Mechanical power of the low-speed shaft in relation to the tip speed ratio.

Figs 15 and 16 show comparisons of the mechanical power of the high-speed and low-speed shafts in relation to the tip speed ratio. The AOSMC controller maintains mechanical power close to the optimal tip speed ratio, ensuring optimal performance. Conversely, the GGSMC controller exhibited inadequate performance with substantial power variations.

**Fig 17:**
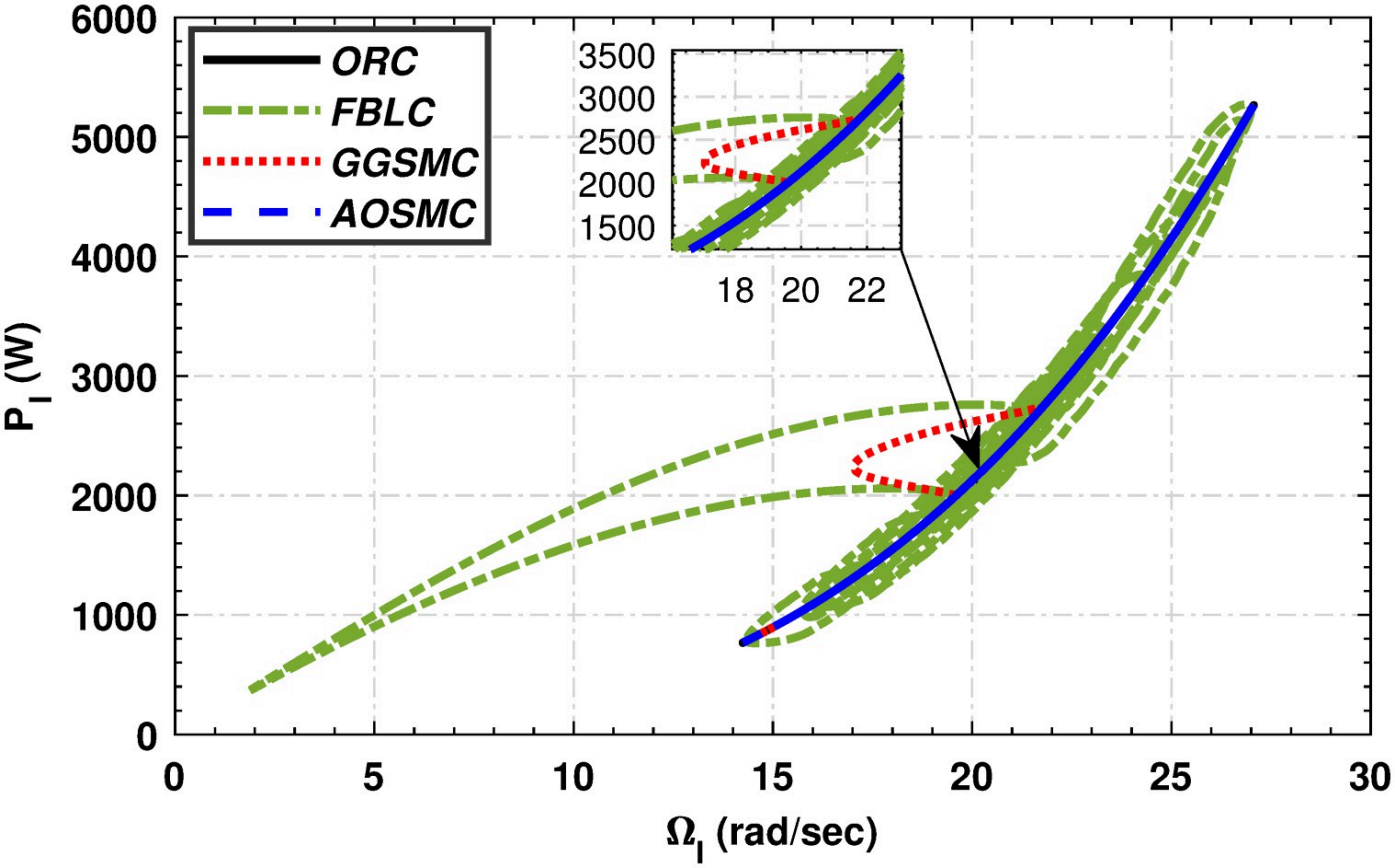
Power variation with respect to the rotational speed of the low-speed shaft.

**Fig 18:**
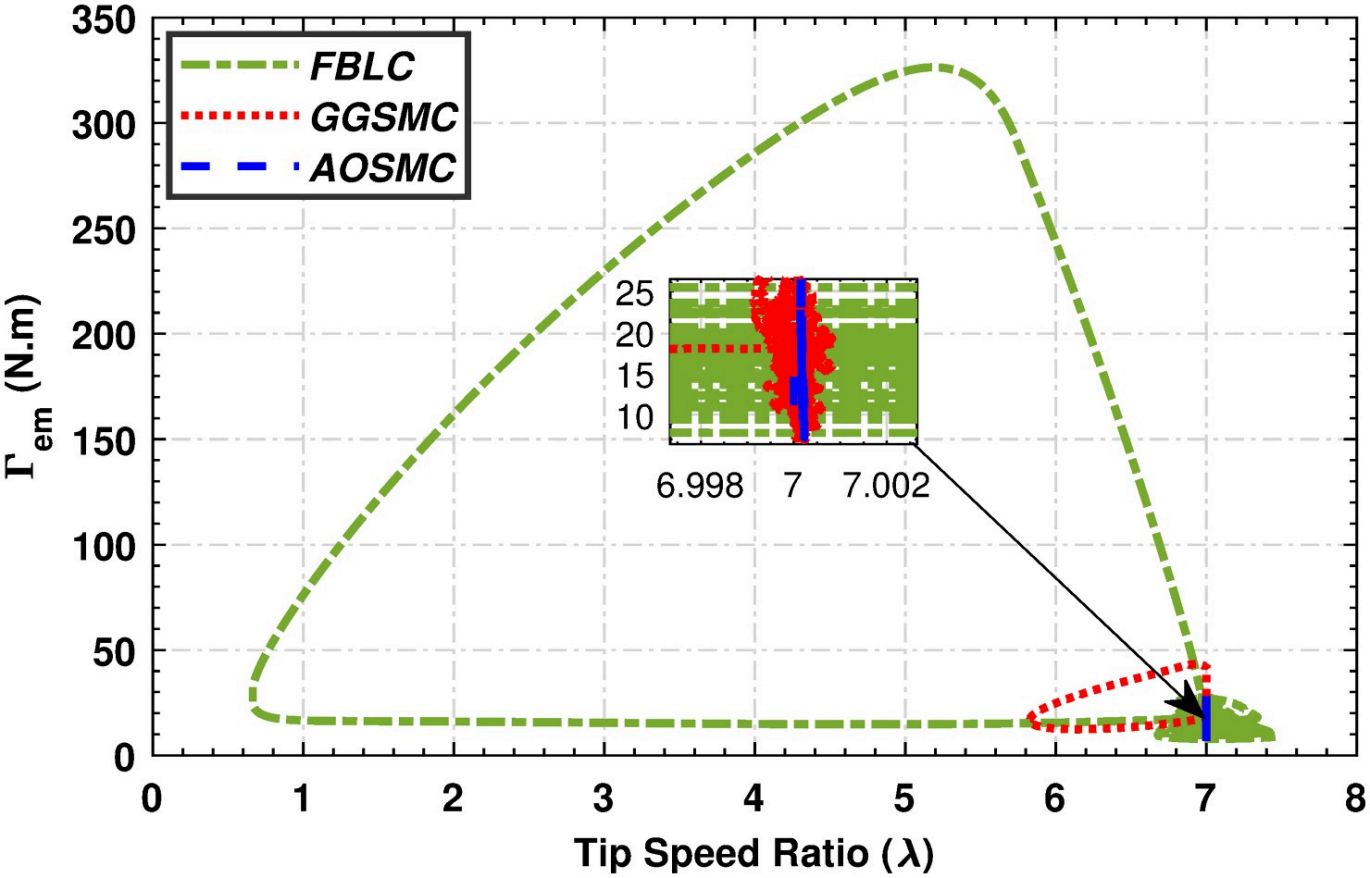
Torque variation with respect to the tip speed ratio.

The relationship between the low-speed shaft rotational speed and the low-speed shaft mechanical power is shown in Fig 17. Evidently, the designed MPPT controllers effectively maintain the VSWT’s rotational speed within the optimal regime characteristic zone, thereby maximizing power extraction. In contrast, the GGSMC controller exhibits significant speed and power variations, indicating inferior performance compared to the other controllers. Fig 18 shows the electromagnetic torque performance of a PMSG with respect to its tip speed ratio (TSR). The magnified area highlights the ability of the AOSMC controller to effectively maintain the electromagnetic torque at the optimal TSR, resulting in maximum power extraction. The AOSMC controller demonstrated the best performance among all controllers, whereas the GGSMC controller exhibited frequent torque fluctuations. Furthermore, the dynamic performance of the MPPT approach is evaluated using four performance metrics: the integral of the time absolute error, the integral of the absolute error, the integral of the time squared error, and the integral of the squared error.

**Fig 19:**
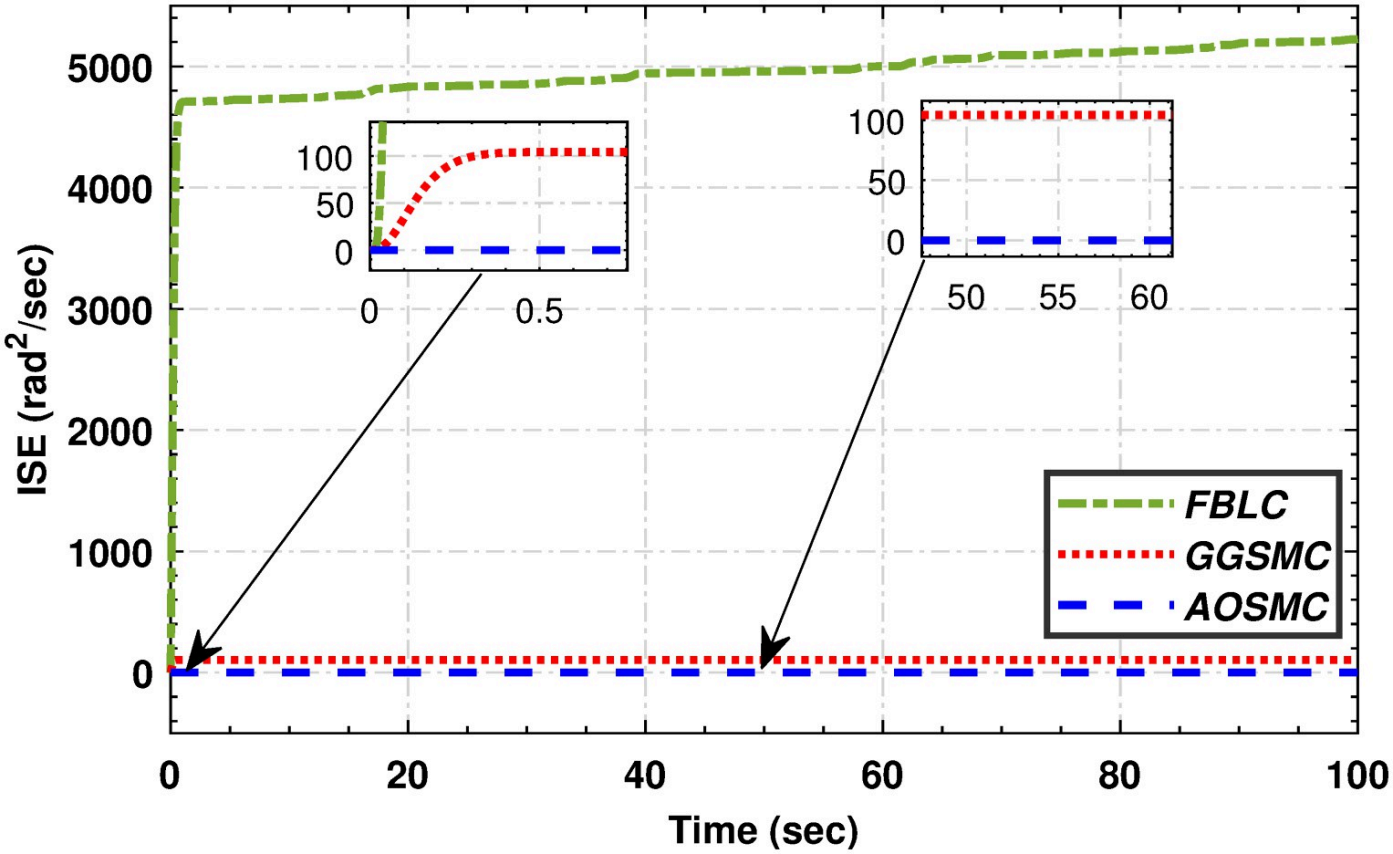
The comparison of integral squared mismatches.

**Fig 20:**
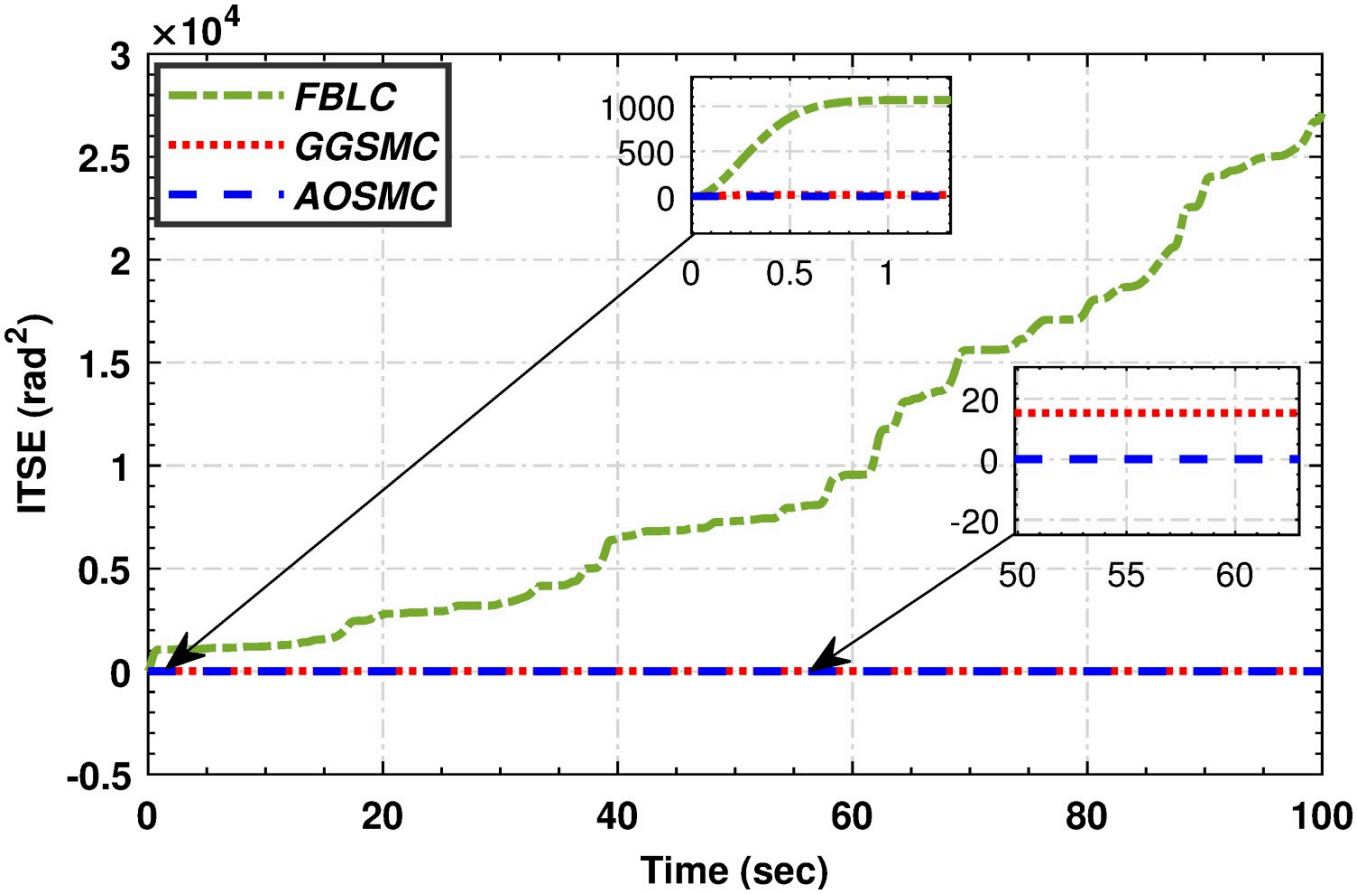
The comparison of integral of time squared mismatch.

**Fig 21:**
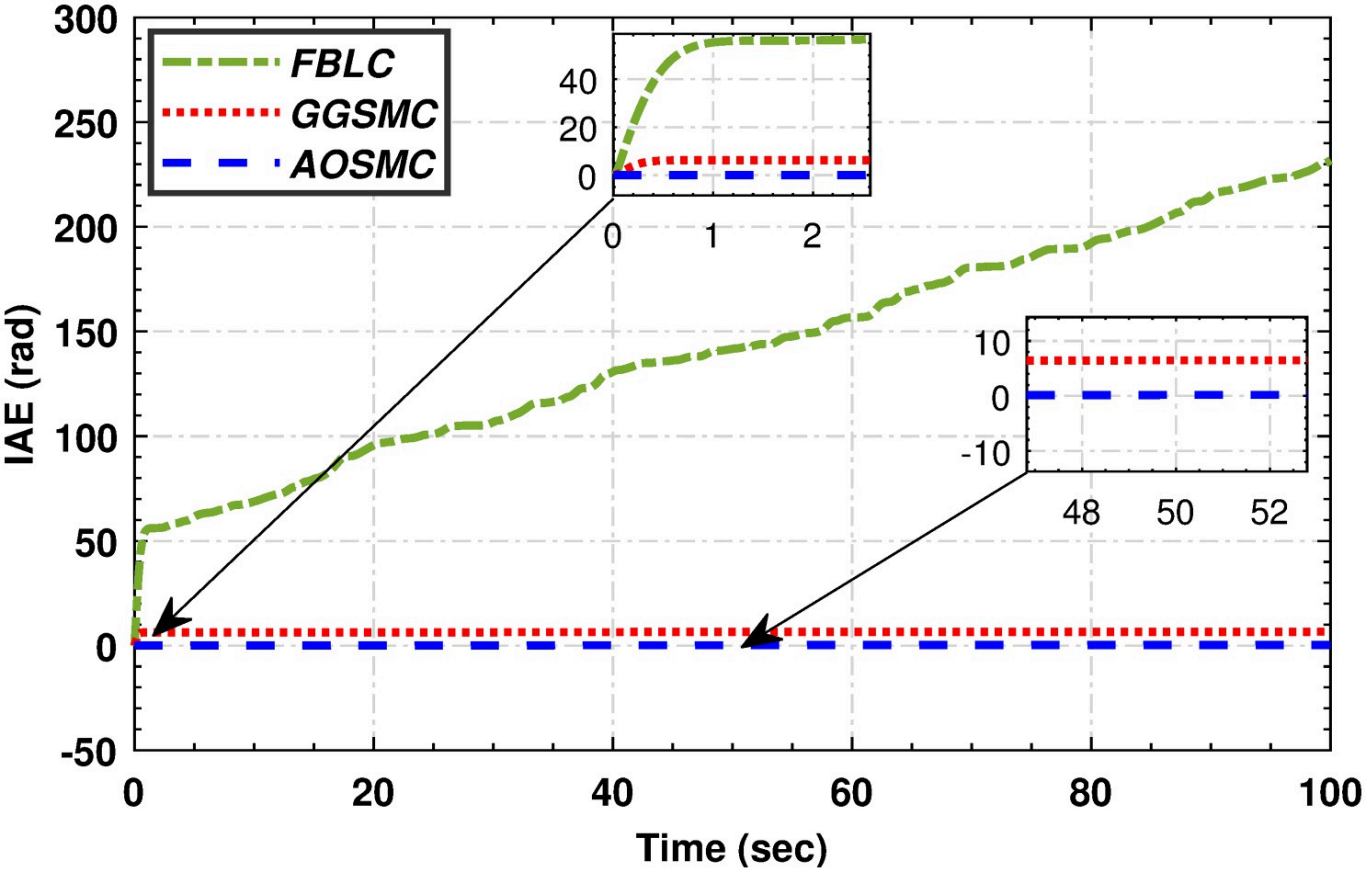
The comparison of integral absolute mismatch.

**Fig 22:**
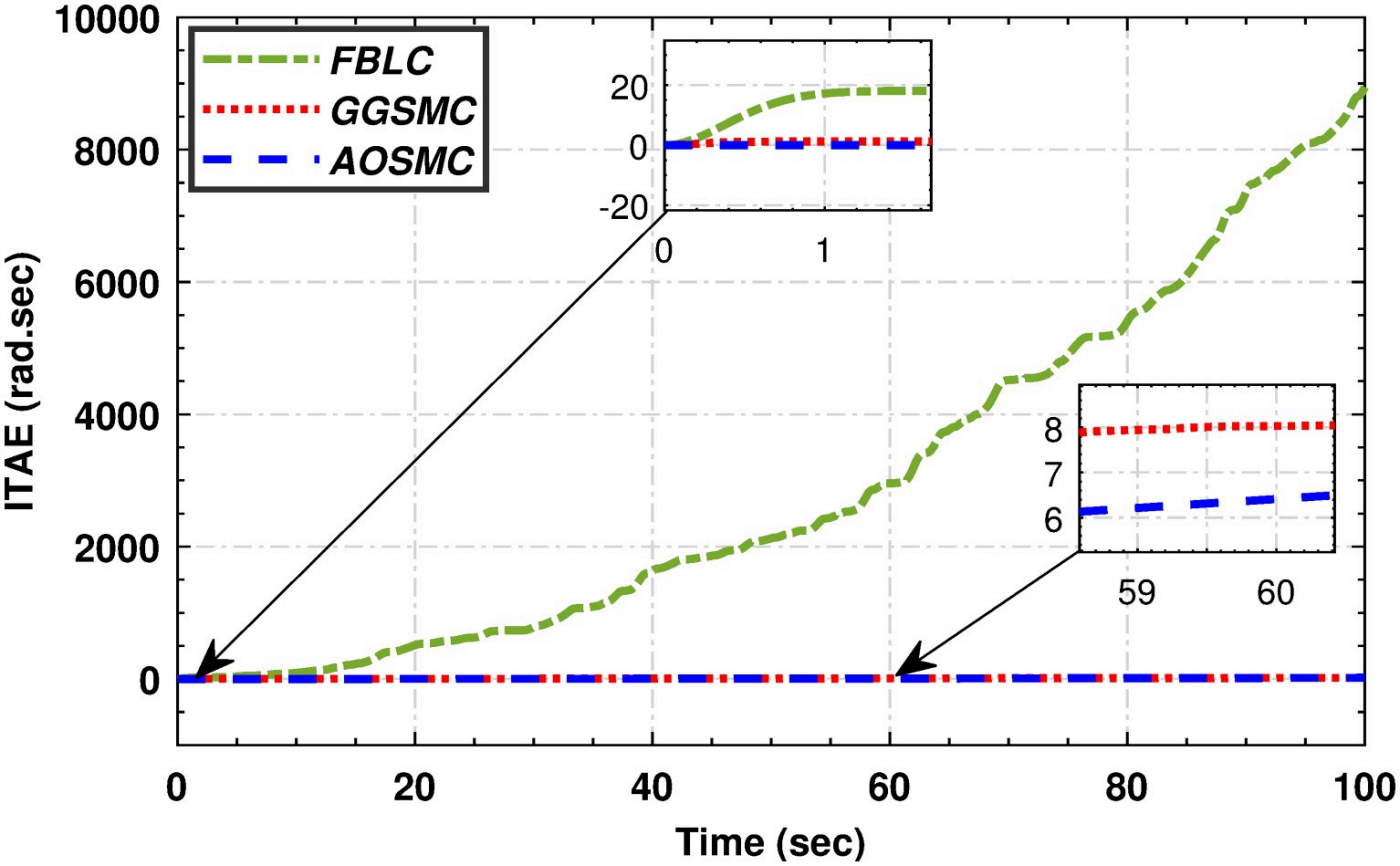
The comparison of integral time absolute mismatch.

Figs 19, 20, 21 and 22 illustrate that the accumulative error of the MPPT controllers decreases over time, indicating their superior performance compared to the GGSMC controller. These metrics provide further evidence of the effectiveness of the AOSMC controller in achieving accurate and stable maximum power point tracking (MPPT).

The comparative analysis strongly supports the superior performance of the AOSMC law compared to the existing approaches, GGSMC, and FBLC. The AOSMC controller accurately and precisely tracked the wind speed, resulting in effective control. It minimizes power fluctuations, efficiently converts, and maintains precise control torque. In addition, the AOSMC outperforms the other controllers in crucial MPPT performance metrics, such as *C*_*p*_ tracking, low-speed shaft mechanical power, high-speed shaft mechanical power, low-speed shaft rotational speed, and electromagnetic torque control. Overall, the proposed AOSMC establishes a new standard for MPPT performance and is a superior choice for wind-energy conversion systems.

## 5 Conclusion

This study used a nonlinear MPPT controller for a standalone 3 kW PMSG-WECS with variable-speed and fixed-pitch configurations. The proposed control strategy combines an arbitrary order sliding mode control design with a high gain differentiator and a feedforward neural network estimator to mitigate chattering phenomena and external disturbances and accurately track the maximum power point. A comparison of the simulation results confirms the superior performance of the proposed control algorithm compared with the conventional feedback linearization and generalized global sliding mode control methods. The integrated components enhance robustness and make the controller more suitable for practical applications, demonstrating its potential for improved MPPT in PMSG-WECS systems. While AOSMC provides substantial advantages in terms of stability and control efficiency, it does come with limitations. These include sensitivity to parameter tuning, complexity, challenges in real-world implementation, potential susceptibility to measurement noise, and the requirement for real-world validation.
